# CteG is a *Chlamydia trachomatis* effector protein that associates with the Golgi complex of infected host cells

**DOI:** 10.1038/s41598-019-42647-3

**Published:** 2019-04-16

**Authors:** Sara V. Pais, Charlotte E. Key, Vítor Borges, Inês S. Pereira, João Paulo Gomes, Derek J. Fisher, Luís Jaime Mota

**Affiliations:** 1 UCIBIO, Departamento de Ciências da Vida, Faculdade de Ciências e Tecnologia, Universidade NOVA de Lisboa, Caparica, Portugal; 2 Department of Microbiology, Southern Illinois University, Carbondale, Illinois USA; 3 Bioinformatics Unit, Department of Infectious Diseases, National Institute of Health, Lisbon, Portugal

## Abstract

*Chlamydia trachomatis* is a bacterial pathogen causing ocular and genital infections in humans. *C*. *trachomatis* multiplies exclusively inside host cells within a characteristic vacuole, from where it manipulates host cells by injecting them with type III secretion effector proteins. Here, we identified CteG as the first *C*. *trachomatis* effector associated with the Golgi. For this, *C*. *trachomatis* strains expressing candidate effectors fused to a double hemagglutinin (2HA) tag were constructed. Then, among these strains, immunofluorescence microscopy revealed that CteG-2HA was delivered into the cytoplasm of infected cells. Between 16–20 h post-infection, CteG-2HA mostly associated with the Golgi; however, CteG-2HA also appeared at the host cell plasma membrane, and at 30 or 40 h post-infection this was its predominant localization. This change in the main localization of CteG-2HA was independent of intact microfilaments or microtubules. Ectopic expression of different regions of CteG (656 amino acid residues) in uninfected cells revealed that its first 100 residues contain a Golgi targeting region. Although a *C*. *trachomatis cteG* mutant did not display a defect in intracellular multiplication, CteG induced a vacuolar protein sorting defect when expressed in *Saccharomyces cerevisiae*. This suggested that CteG might function by subverting host cell vesicular transport.

## Introduction

*Chlamydia trachomatis* serovars are obligate intracellular bacterial pathogens usually causing ocular and genital infections that affect millions of people worldwide and can lead to blindness and sterility. Serovars A-C are normally associated with trachoma^1^, while serovars D-K are the most common cause of sexually transmitted bacterial infections^2^. The less common serovars L1–L3 cause lymphogranuloma venereum (LGV), an invasive infection^2^. The *Chlamydia* genus includes other species pathogenic for humans (*C*. *pneumoniae* and *C*. *psittaci*) and other animals (*C*. *abortus*, *C*. *avium*, *C*. *caviae*, *C*. *felis*, *C*. *gallinacea*, *C*. *muridarum*, *C*. *pecorum*, *C*. *psittaci*, and *C*. *suis*)^3^.

*Chlamydia* are characterized by a developmental cycle involving an infectious but non-replicative form, the elementary body (EB), and a non-infectious but replicative form, the reticulate body (RB). Adherence of extracellular EBs to host cells leads to invasion and formation of a membrane-bound vacuolar compartment (known as the inclusion) where *Chlamydia* resides, develops and grows intracellularly^4^. As with many other Gram-negative bacteria^5^, the capacity of *Chlamydia* to subvert host cells largely relies on a type III secretion (T3S) system mediating the transport of effector proteins into host cells^6^. In general, the biological function of T3S effectors depends on their biochemical activity, timing of delivery and specific subcellular targeting in host cells, and is coordinated with the action of other effectors injected by the same bacterium^7,8^. In *Chlamydia*, T3S effectors include the Inc proteins, characterized by a bilobed hydrophobic motif mediating their insertion into the inclusion membrane^9,10^. The identification of *Chlamydia* effectors without the bilobed hydrophobic motif is normally more challenging because their primary structure normally lacks other obvious distinguishable features. However, several of these non-Inc *C*. *trachomatis* T3S effectors (e.g., TarP, TepP, CT694/TmeA) have been identified and shown to modulate chlamydial invasion and diverse host cell functions^4,11–15^. There are also *Chlamydia* effectors, such as deubiquitinating enzymes^16,17^, which localize within the cytoplasm of host cells and that have not been shown to be T3S substrates, as well as chlamydial T3S substrates secreted into the inclusion lumen^18,19^. Some of the non-Inc chlamydial effectors localize at the inclusion membrane^17,20–22^, at the host cell plasma membrane^22^, or at the host cell nucleus^23–25^, while others are membrane-associated^11,26^ or have undefined localization.

In this work, following the identification of candidate chlamydial T3S substrates using *Yersinia enterocolitica* as a heterologous host^27,28^, we show that the *C*. *trachomatis* CT105 protein (CTL0360 in *C*. *trachomatis* serovar L2 strain 434/Bu; L2/434) is delivered into host cells during infection. In infected cells, bacterially-delivered CT105 initially mainly localized at the Golgi complex and then at the plasma membrane. CT105 is the first *Chlamydia* protein described to localize at the Golgi in infected cells, and we identified a Golgi-targeting region within its first 100 amino acid residues. Using *Saccharomyces cerevisae* as model, we also show that CT105 can modulate eukaryotic vesicular trafficking.

## Results

### CT105-2HA is delivered by *C*. *trachomatis* into the cytoplasm of infected cells

**Figure 1:**
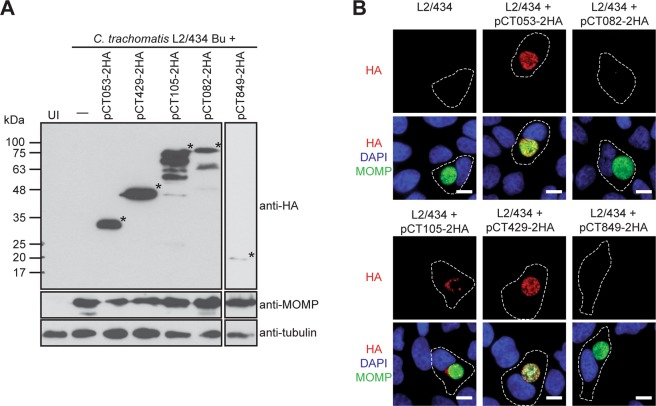
The chlamydial candidate T3S effector CT105 is delivered by *C*. *trachomatis* into the cytoplasm of infected cells. HeLa cells were either left uninfected (UI) or infected by *C*. *trachomatis* L2/434-derived strains harboring the indicated plasmids, encoding candidate T3S effectors (CT053, CT429, CT105, CT082, CT849) with a 2HA epitope tag at their C-termini. (**A**) At 40 h p.i., whole cell extracts were analyzed by immunoblotting with antibodies against HA, MOMP (bacterial loading control) and α-tubulin (HeLa loading control) using SuperSignal West Pico detection kit (Thermo Fisher Scientific), or SuperSignal West Femto detection kit (Thermo Fisher Scientific) for CT849-2HA. Asterisks indicate the bands likely corresponding to the full-length proteins. Whole immunoblots are presented in Supplementary Fig. S1. (**B**) At 20 h p.i., cells were fixed with 4% (w/v) PFA, stained with DAPI (blue), immunolabeled with antibodies against HA (red) and *C*. *trachomatis* MOMP (green), and appropriate fluorophore-conjugated secondary antibodies, and imaged by fluorescence microscopy. Scale bars, 10 µm.

To test if the candidate chlamydial T3S substrates CT053, CT082, CT105, CT429, and CT849^27,28^ can be transported by *Chlamydia* into the cytoplasm of host cells, *C*. *trachomatis* strain L2/434 was transformed with plasmids encoding these proteins with a double hemagglutinin (2HA) epitope tag at their C-termini. Protein production was confirmed by immunoblotting of extracts of HeLa cells infected for 40 h with *C*. *trachomatis* strains harboring plasmids encoding CT053-2HA (predicted molecular mass of 17 kDa), CT082-2HA (60 kDa), CT105-2HA (68 kDa), CT429-2HA (39 kDa), or CT849-2HA (18 kDa) (Figs 1A and S1). The strains producing CT053-2HA, CT082-2HA and CT105-2HA also showed species migrating on SDS-PAGE at a molecular mass different from the one predicted for the full-length proteins (Figs 1A and S1), as previously observed when identical 2HA-tagged versions of the proteins were produced in *Y*. *enterocolitica*^27,28^. Overall, these experiments confirmed that the constructed *C*. *trachomatis* strains expressed the expected 2HA-epitope tagged proteins.

To analyze the subcellular localization of CT053-2HA, CT082-2HA, CT105-2HA, CT429-2HA, and CT849-2HA, HeLa cells were infected for 20 or 40 h with *C*. *trachomatis* L2/434-derived strains bearing the corresponding encoding plasmids and then analyzed by immunofluorescence microscopy. At 20 p.i. (Fig. 1B) or 40 h p.i. (Supplementary Fig. S2), CT053-2HA, CT082-2HA, CT429-2HA, and CT849-2HA were only detected within the inclusion and/or colocalizing with the *C*. *trachomatis* major outer membrane protein (MOMP) signal (Figs 1B and S2). In contrast, CT105-2HA was detected outside of the inclusion, indicating it is bacterially-delivered into the cytoplasm of infected host cells (Figs 1B and S2).

### Full-length orthologues of CT105 are only present in *C*. *muridarum* and *C*. *suis*, and *ct105* expression is mostly restricted to *C*. *trachomatis* LGV strains

CT105 is a protein of 656 amino acid residues whose sequence does not show significant similarity to other proteins except for potential orthologues in other *Chlamydia* spp. However, full-length orthologues of CT105 were only found in *C*. *muridarum* and *C*. *suis* (Supplementary Table S1). In *C*. *suis* and in other *Chlamydia* spp. different open reading frames might encode proteins with some identity (between 30–22%) to only some parts of the amino acid sequence of CT105 (Supplementary Table S1). As previously noted^29^, analysis of the mRNA levels of *ct105* by reverse transcription quantitative PCR (RT-qPCR) in different *C*. *trachomatis* strains revealed that the gene is only significantly expressed in LGV strains (serovars L1–L3) (Supplementary Fig. S3). Furthermore, also as previously noted^29^, considering the promoter region of *ct105* based on the transcription start site identified in *C*. *trachomatis* LGV strain L2b/UCH-1/proctitis^30^, *C*. *trachomatis* non-LGV strains (serovars A-K) lack 74 nucleotides upstream from the putative −10 region recognized by *C*. *trachomatis* σ^66^ (Supplementary Fig. S4). It has also been previously shown that *ct105* is a pseudogene in *C*. *trachomatis* ocular strains (serovars A-C)^31^. Therefore, among *C*. *trachomatis* serovars, active CT105 is mostly produced by LGV strains.

### Characterization of *C*. *trachomatis* expressing plasmid-encoded CT105-2HA

The L2/434 strain harboring pCT105-2HA, was then characterized by comparison to the parental strain. The profile of expression of *ct105* during the developmental cycle of *C*. *trachomatis* was similar between the two strains (Supplementary Fig. S5). In agreement with previous observations^27^ (Supplementary Fig. S3), the highest levels of *ct105* mRNA were detected at 2 h p.i. (Supplementary Fig. S5). Furthermore, at 2 h p.i., the strain harboring pCT105-2HA showed a 10-fold increase in the mRNA levels of *ct105* relative to the parental strain (Supplementary Fig. S5). However, expression of plasmid-encoded CT105-2HA in *C*. *trachomatis* had no significant impact on chlamydial growth (Supplementary Fig. S5).

In extracts of HeLa cells infected by *C*. *trachomatis* L2/434 harboring pCT105-2HA, the 2HA-tagged protein could be detected by immunoblotting from 16 to 40 h p.i. (Supplementary Fig. S6). In addition to the band likely corresponding to full-length CT105-2HA (68 kDa), several faster migrating species of a lower molecular mass were consistently observed between 20 to 40 h p.i. (Supplementary Fig. S6). Similar observations were made by immunoblotting of extracts of HeLa cells infected by strain L2/434 harboring a plasmid (pTet-CT105-2HA) where production of CT105-2HA is driven by the tetracycline promoter (P_*tet*_) (Supplementary Fig. S6). This indicates that plasmid-encoded CT105-2HA is modified and/or degraded as the *C*. *trachomatis* developmental cycle progresses. Immunoblotting of extracts of HeLa cells infected by L2/434 harboring either pCT105-2HA or pTet-CT105-2HA, or of corresponding *Chlamydia*-enriched extracts, suggested that this modification and/or degradation of CT105-2HA occurs within the bacterium (Supplementary Fig. S6).

### CT105-2HA localizes at the Golgi complex in *C*. *trachomatis*-infected cells

To analyze in further detail the localization of CT105-2HA during infection, HeLa cells were infected for different times by *C*. *trachomatis* L2/434 harboring pCT105-2HA and then analysed by immunofluorescence microscopy. At 2, 4, and 8 h p.i., CT105-2HA could be detected colocalizing with the signal for *C*. *trachomatis* Hsp60 (Supplementary Fig. S7), which suggested its presence within the bacterial cells. At 16 and 20 h p.i., CT105-2HA was detected outside of the inclusion in approximately 37% and 90% of the infected cells, respectively (Supplementary Fig. S7); at 30 and 40 h p.i., the protein was found outside of the inclusion in all infected cells (Supplementary Fig. S7). While at 16, 20, and 30 h p.i. CT105-2HA was detected in the host cell cytoplasm concentrated at one of the sides of the inclusion, at 30 h p.i. the protein was also detected at the periphery of the infected cells (Supplementary Fig. S7). Furthermore, at 40 h p.i., CT105-2HA was mostly seen at the periphery of the infected cells (Supplementary Fig. S7).

**Figure 2:**
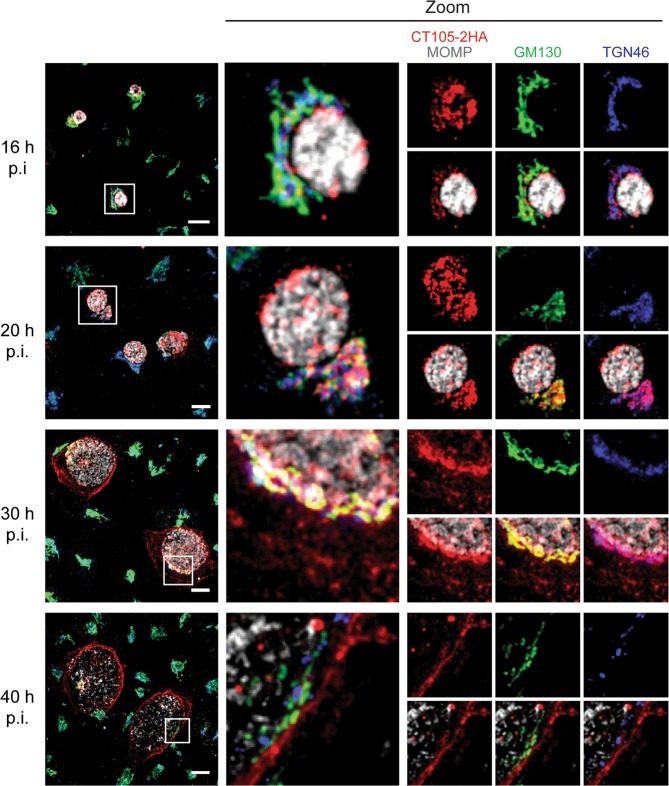
CT105 colocalizes with the Golgi complex in *C*. *trachomatis*-infected cells. HeLa cells were infected by *C*. *trachomatis* L2/434 encoding CT105-2HA. At the indicated times p.i., cells were fixed with 4% (w/v) PFA, immunolabeled with antibodies against HA (red), *C*. *trachomatis* MOMP (gray), GM130 (green), and TGN46 (blue), and appropriate fluorophore-conjugated secondary antibodies. In the area delimited by a white square (left-side panels) images were zoomed (middle and right-side panels). Immunolabeled cells were examined by confocal fluorescence microscopy, and images correspond to single *z* sections. Scale bars, 10 µm.

The accumulation of CT105-2HA near the inclusion in the cytoplasm of infected cells suggested it could localize at the Golgi complex. This immunofluorescence signal of CT105-2HA also appeared less compact at longer times of infection, which was evocative of the Golgi fragmentation seen in *C*. *trachomatis* infected-cells^32^. To analyze this, HeLa cells infected by *C*. *trachomatis* L2/434 harboring pCT105-2HA were fixed at 16, 20, 30, or 40 h p.i. and then analyzed by immunofluorescence microscopy. This revealed that CT105-2HA localized in the Golgi region at 16, 20, and 30 h p.i., and that this was much less evident at 40 h p.i. (Fig. 2). There was not a perfect colocalization of CT105-2HA with *cis*-Golgi (GM130) and TGN (TGN46) markers, but the immunofluorescence signal of CT105-2HA near the inclusion accompanied the dispersion of the Golgi complex during infection (Fig. 2).

**Figure 3:**
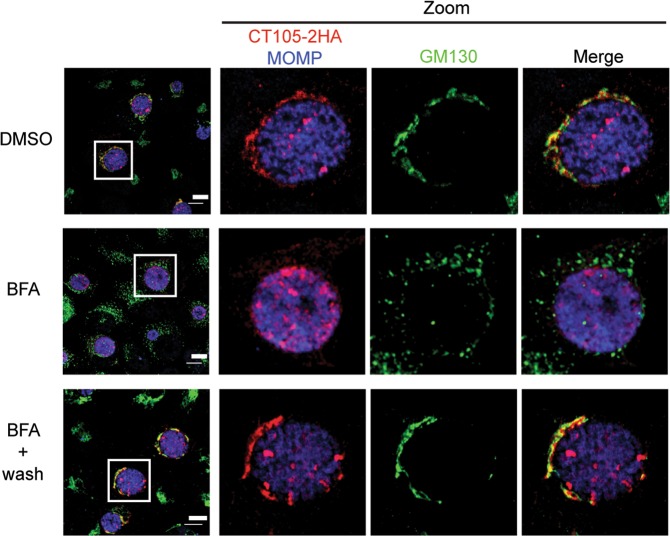
CT105 associates with the Golgi complex in *C*. *trachomatis*-infected cells. HeLa cells were infected by *C*. *trachomatis* L2/434 encoding CT105-2HA. At 19 h p.i., cells were treated for 1 h with DMSO or 1 µg/ml BFA. Then, the cells were either fixed with 4% (w/v) PFA (upper and middle panels) or washed with complete medium lacking BFA and incubated for an additional 1 h and then fixed with 4% (w/v) PFA (lower panels). Fixed cells were immunolabeled with antibodies against HA (red), *C*. *trachomatis* MOMP (gray), and GM130 (green), and appropriate fluorophore-conjugated secondary antibodies. In the area delimited by a white square (left-side panels) images were zoomed (right-side panels). All immunolabeled cells were examined by confocal fluorescence microscopy, and images correspond to single *z* sections. Scale bars, 10 µm.

To further confirm the association of CT105-2HA with the Golgi, HeLa cells infected by the L2/434 strain harboring pCT105-2HA were treated with Brefeldin A (BFA), which induces reversible Golgi fragmentation^33^. For this, at 19 h p.i., the infected cells were either incubated for 1 h with BFA or with dimethyl sulfoxide (DMSO)-solvent alone and then fixed. In addition, the infected cells were incubated for 1 h with BFA, washed out from the BFA, incubated for 1 h and then fixed. Immunofluorescence microscopy revealed that in the DMSO-treated infected cells, CT105-2HA accumulated in the Golgi region (Fig. 3), similarly to untreated cells infected for 16 or 20 h p.i. (Fig. 2). In cells treated with BFA, the GM130-labeled Golgi fragmented into small vesicles and the immunofluorescence signal of CT105-2HA became dispersed and mostly below the limit of detection (Fig. 3). In cells treated with BFA and subsequently washed-out of the drug, both the GM130-labeled Golgi and CT105-2HA appeared mostly compact and near the inclusion (Fig. 3). Overall, this showed that CT105-2HA associates with the Golgi, at 16–20 h p.i., upon the initial detection of its delivery by *C*. *trachomatis* into the cytoplasm of infected host cells.

### CT105-2HA localizes at the host cell plasma membrane in *C*. *trachomatis*-infected cells

**Figure 4:**
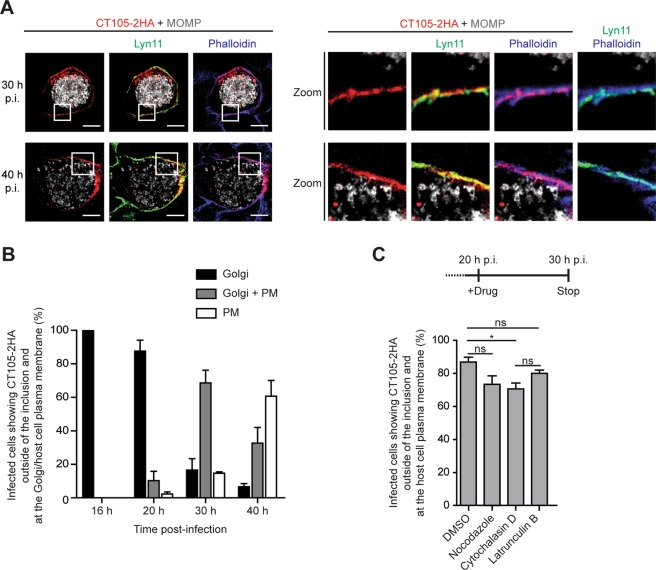
CT105 localizes at plasma membrane in *C*. *trachomatis*-infected cells. HeLa cells were infected by *C*. *trachomatis* L2/434 encoding pCT105-2HA. (**A**) Cells were also transfected with a plasmid encoding the plasma membrane marker Lyn11-mEGFP (green). At 30 or 40 h p.i., cells were fixed with 4% (w/v) PFA, stained with phalloidin (blue), and immunolabeled with antibodies against HA (red) and *C*. *trachomatis* MOMP (gray), and appropriate fluorophore-conjugated secondary antibodies. Cells were imaged by confocal fluorescence microscopy and images correspond to single *z* sections. In the area delimited by a white square (left-side panels) images were zoomed (right-side panels). Scale bars, 10 µm. (**B**) Infected cells were fixed with 4% (w/v) PFA at 16, 20, 30 or 40 h p.i. and immunolabeled with antibodies against HA, *C*. *trachomatis* MOMP, and GM130, and appropriate fluorophore-conjugated secondary antibodies. Fluorescence microscopy was then used to enumerate cells showing CT105-2HA only at the Golgi, only at the plasma membrane (PM), or both at the Golgi and at the plasma membrane (Golgi + PM). Data are mean ± standard error of the mean (SEM) of three independent experiments (n ≥ 25). (**C**) At 20 h p.i., infected cells were treated with DMSO, 1 µg/ml nocodazole, 2 µM cytochalasin D or 500 nM latrunculin B. At 30 h p.i., the cells were fixed with 4% (w/v) PFA and labeled with immunolabeled with antibodies against HA, *C*. *trachomatis* MOMP, and appropriate fluorophore-conjugated secondary antibodies. Fluorescence microscopy was used to enumerate cells showing CT105-2HA at the periphery of the cell/plasma membrane. Data are mean ± SEM of three independent experiments (n = 50). P-values were obtained by one-way ANOVA and Bonferroni post-test analyses; *statistical significant (P < 0.05); ns, not significant.

CT105-2HA was also detected in the periphery of host cells infected by *C*. *trachomatis* at 30 or 40 h p.i. (Supplementary Fig. S7), which suggested it could localize at the plasma membrane. To analyze this, HeLa cells were infected for 30 and 40 h p.i. by *C*. *trachomatis* L2/434 harboring CT105-2HA and transfected with a plasmid encoding a palmitoylated/myristoylated peptide from Lyn kinase (Lyn11) [targeting proteins to the plasma membrane^34^] fused to monomeric EGFP^35^ (Lyn11-mEGFP). The cells were then fixed and analyzed by immunofluorescence microscopy. Although CT105-2HA did not show a perfect colocalization with either Lyn11-mEGFP or with phalloidin-stained cortical actin (Fig. 4A), this showed that at 30 and 40 h p.i., CT105-2HA localizes at the host cell plasma membrane.

The number of infected cells showing CT105-2HA outside of the inclusion and at the Golgi or at the plasma membrane were then enumerated (Fig. 4B), which confirmed that in *C*. *trachomatis*-infected cells, initial delivery of CT105-2HA into the host cell cytoplasm (first detected at 16 h p.i.) results in its accumulation at the Golgi region, but as infection progresses this localization of the protein becomes less frequent and CT105-2HA accumulates more often at the periphery of the cell. These observations were recapitulated by the analysis of HeLa cells infected by *C*. *trachomatis* L2/434 harboring pTet-CT105-2HA (Supplementary Fig. S8). To test if CT105-2HA was transported from the Golgi to the plasma membrane in vesicles associated with the microtubule and/or actin cytoskeleton, HeLa cells were infected with *C*. *trachomatis* harboring pCT105-2HA. At 20 h p.i., when CT105-2HA mostly localizes at the Golgi region (Fig. 4B), the cells were incubated in the presence of DMSO solvent as a control, cytochalasin D or latrunculin B (to inhibit actin polymerization by different mechanisms), or nocodazole (to depolymerize microtubules) (Fig. 4C). The cells were then fixed at 30 h p.i., when CT105-2HA mostly localizes at the plasma membrane (Fig. 4B), and analyzed by immunofluorescence microscopy. We observed a statistically significant difference in the localization of CT105-2HA at the cell periphery when microfilaments were disrupted with cytochalasin D (Fig. 4C). However, the small difference relative to DMSO-treated cells, and the absence of a significantly different effect upon disruption of the microfilaments with latrunculin B (Fig. 4C), suggests this should have no biological significance. Furthermore, disruption of the microtubule network also did not significantly affect the localization of CT105-2HA at the cell periphery (Fig. 4C). Therefore, CT105-2HA changes its predominant localization during infection of host cells by *C*. *trachomatis* from the Golgi to the plasma membrane, but this is independent of intact host cell microfilaments and microtubules.

### The first 100 amino acid residues of CT105 are sufficient to target the protein to the Golgi in uninfected mammalian cells

**Figure 5:**
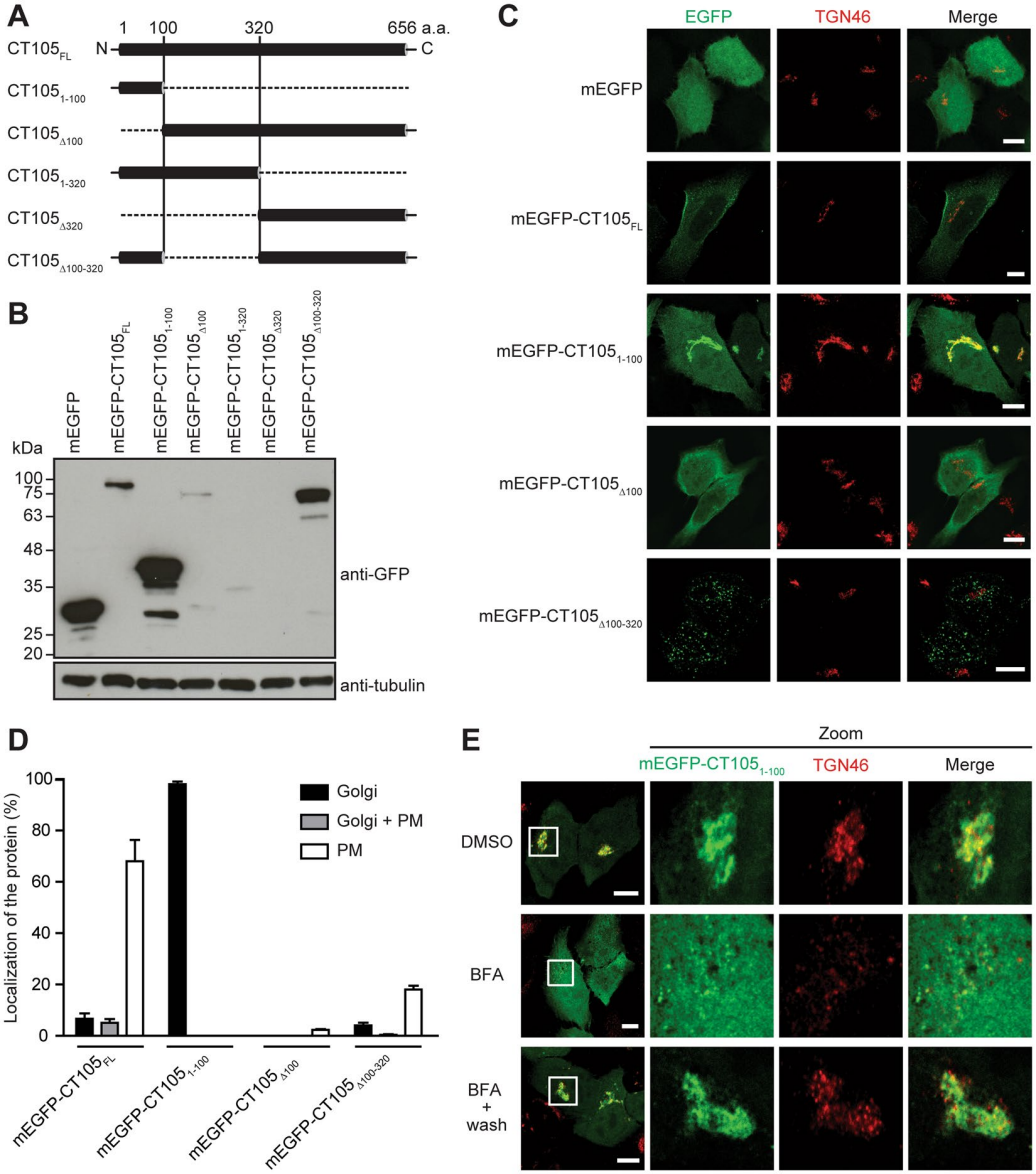
The first 100 amino acids of CT105 contain a Golgi-targeting region. HeLa cells were transfected for 24 h with plasmids encoding monomeric EGFP (mEGFP) or different regions of CT105 fused to the C-terminus of mEGFP (CT105-mEGFP proteins), as indicated. (**A**) Schematic representation of the mEGFP-CT105 proteins expressed and analyzed. (**B**) Total extracts of transfected cells were analyzed by immunoblotting with antibodies against GFP and α-tubulin (HeLa loading control) using SuperSignal West Pico detection kit (Thermo Fisher Scientific). (**C**) Transfected cells were fixed with 4% (w/v) PFA, labeled with antibodies against TGN46 (red), and the appropriate fluorophore-conjugated secondary antibody, and imaged by confocal fluorescence microscopy. Images correspond to single *z* sections. Scale bars, 10 µm. (**D**) Cells immunolabeled as described in C were enumerated by fluorescence microscopy for localization of mEGFP-CT105 only at the Golgi, only at the plasma membrane (PM), or both at the Golgi and plasma membrane (Golgi + PM). Data are mean ± standard error of the mean of three independent experiments (n = 100). (**E**) Cells were treated for 1 h with DMSO or 1 µg/ml BFA. Then, the cells were either fixed with 4% (w/v) PFA (upper and middle panels) or washed with complete medium lacking BFA and incubated for an additional 1 h and then fixed with 4% (w/v) PFA (lower panels). The fixed cells were immunolabeled with antibodies against TGN46 (red), and the appropriate fluorophore-conjugated secondary antibody, and examined by confocal immunofluorescence microscopy. Images correspond to single *z* sections. In the area delimited by a white square (left-side panels) images were zoomed (right-side panels). Scale bars, 10 µm.

To map the regions of CT105 that could determine its different localizations within the cytoplasm of mammalian cells, HeLa cells were first transfected for 24 h with plasmids encoding full length CT105 fused to the C- or N-termini of monomeric EGFP (mEGFP-CT105_FL_ and CT105_FL_-mEGFP, respectively) or with C-terminal 2HA-epitope tag. Immunoblotting of whole cell extracts confirmed the production of proteins of the predicted molecular mass (Supplementary Fig. S9). Furthermore, fluorescence microscopy showed that mEGFP-CT105_FL_, CT105_FL_-mEGFP, or CT105-2HA localized predominantly at the periphery of the cell (Supplementary Fig. S9), recapitulating a main localization of CT105-2HA in cells infected by *C*. *trachomatis* for 30 h or 40 h (Fig. 4B). Next, HeLa cells were transfected with plasmids encoding mEGFP-CT105_FL_ (predicted molecular mass of 96 kDa) and different truncated versions of CT105 fused to the C-terminus of mEGFP: mEGFP-CT105_1–100_ (38 kDa), mEGFP-CT105_Δ100_ (86 kDa) mEGFP-CT105_1–320_ (60 kDa), mEGFP-CT105_Δ320_ (65 kDa), or mEGFP-CT105_Δ100–320_ (74 kDa) (Fig. 5A). As the primary structure of CT105 revealed no predicted transmembrane domains or other obvious targeting motifs, these truncations were designed only considering the predicted secondary structure of CT105 [deduced using JPred4^36^]. Immunoblotting of whole cell extracts confirmed the production of mEGFP-CT105_FL_, mEGFP-CT105_1–100_, mEGFP-CT105_Δ100_, or mEGFP-CT105_Δ100–320_ proteins of the predicted molecular mass (Fig. 5B). The production of mEGFP-CT105_1–320_ and mEGFP-CT105_Δ320_ proteins of the predicted molecular mass was not detected (Fig. 5B), and they were not further analyzed. HeLa cells ectopically expressing mEGFP-CT105_FL_, mEGFP-CT105_1–100_, mEGFP-CT105_Δ100_, or mEGFP-CT105_Δ100–320_ were fixed and analyzed by immunofluorescence microscopy for localization of the fusion protein at the cell periphery (as an indication of plasma membrane localization) and at the Golgi. The mEGFP-CT105_FL_ protein localized only at the cell periphery in 68 ± 8% of the transfected cells, and only at the Golgi in 7 ± 2% of the transfected cells (Fig. 5C,D). However, the fluorescent signal was generally weak in the Golgi region (Fig. 5C). Strikingly, mEGFP-CT105_1–100_ localized at the Golgi region in almost all transfected cells (98 ± 1%) and was not detected at the cell periphery (Fig. 5C,D). In contrast, mEGFP-CT105_Δ100_ was mostly cytosolic and was never seen at the Golgi complex and only seldom (2 ± 0%) at the cell periphery (Fig. 5C,D). mEGFP-CT105_Δ100–320_ showed a punctate/vesicular appearance with no marked localization at the Golgi (4 ± 1%) or cell periphery (18 ± 1%), particularly when compared with mEGFP-CT105_Δ100_ or mEGFP-CT105_FL_ (Fig. 5C,D).

To confirm the association of mEGFP-CT105_1–100_ with the Golgi, and to analyze its localization within the Golgi complex, HeLa cells that had been transfected for 24 h were either incubated for 1 h with BFA or DMSO solvent, and then fixed. In addition, the transfected cells were incubated for 1 h with BFA, washed out from the BFA, incubated for 1 h, and fixed. Immunofluorescence microscopy revealed that in the control DMSO-treated cells, mEGFP-CT105_1–100_ localized at the Golgi region, and showed significant colocalization with TGN46 (Fig. 5E) but not with GM130 (Supplementary Fig. S9). BFA-induced Golgi fragmentation resulted in near complete dispersion of the fluorescence signal of mEGFP-CT105_1–100_ (Figs 5E and S9). However, BFA-induced Golgi fragmentation followed by BFA wash out revealed again a compact Golgi complex and mEGFP-CT105_1–100_ in that region mostly colocalizing with TGN46 (Fig. 5E) but not with GM130 (Supplementary Fig. S9).

In summary, ectopically expressed full-length CT105 (mEGFP-CT105_FL_, CT105_FL_-mEGFP, and CT105-2HA) localized at the cell periphery and mEGFP-CT105_1–100_ at the Golgi, generally recapitulating the localization of CT105-2HA in infected cells. As mEGFP-CT105_1–100_ showed a striking association with the TGN in uninfected cells, this indicated that the first 100 amino acids of CT105 contain a Golgi-targeting region.

### CT105 is not essential for intracellular multiplication of *C*. *trachomatis* in infected tissue culture cells

**Figure 6:**
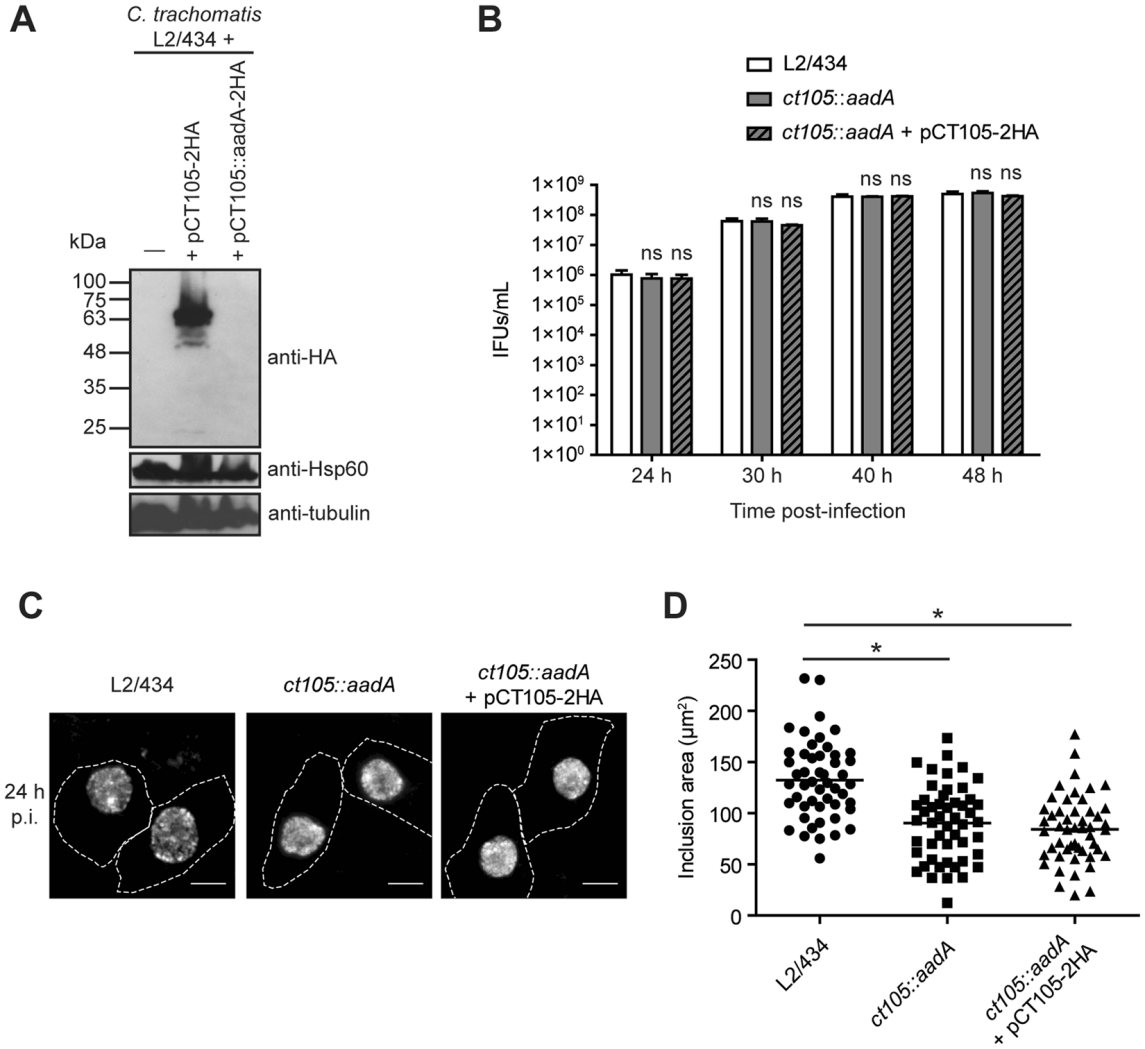
A *C*. *trachomatis ct105::aadA* insertional mutant is not defective for intracellular growth in tissue culture cells. (**A**,**C**) *trachomatis ct105*::*aadA* insertional mutant was generated in the strain L2/434 by the targeted insertion of a modified group II intron carrying a spectinomycin-resistance gene. (**A**) Hela cells were infected by *C*. *trachomatis* L2/434 harboring a plasmid encoding CT105-2HA or by an identical plasmid carrying a *ct105*::*aadA*-2HA mutant allele. Whole cell extracts were analyzed by immunoblotting with antibodies against HA, *C*. *trachomatis* Hsp60 (bacterial loading control) and α-tubulin (HeLa loading control) using SuperSignal West Pico detection kit (Thermo Fisher Scientific) for Hsp60 and α-tubulin, or SuperSignal West Femto detection kit (Thermo Fisher Scientific) for HA. (**B**) HeLa cells were infected for 24, 30, or 40 h by *C*. *trachomatis* L2/434 or *ct105::aadA* mutant at the multiplicity of infection of 1, and recoverable inclusion forming units (IFUs) were enumerated. Data ± standard error of the mean of 3 independent experiments. For each time-point, P-values were obtained by one-way ANOVA and Dunnett post-test analyses relative to the L2/434 parental strain; ns, not significant (P ≥ 0.05). (**C**) Hela cells were infected for 24 h by *C*. *trachomatis* L2/434, *ct105::aadA* mutant, or *ct105:aadA* harboring pCT105-2HA. Cells were fixed with methanol, labeled with goat anti-*C*. *trachomatis* FITC-conjugated antibody and imaged by fluorescence microscopy. Scale bars, 10 µm. (**D**) The inclusion area was measured (from images as those depicted in C) for 50 particles randomly chosen from independent images using Fiji software. P-values were obtained by one-way ANOVA and Dunnett post-test analyses; *statistical significant (P < 0.05).

To examine the importance of CT105 during infection of tissue culture cells by *C*. *trachomatis*, a L2/434-derived strain where the *ct105* gene was inactivated by an insertion between nucleotides 261 and 262 of a modified group II intron containing a spectinomycin-resistance gene (*aadA*) was constructed (Supplementary Fig. S10). To confirm that insertion of the intron prevented production of CT105, in the absence of a specific anti-CT105 antibody, a *C*. *trachomatis* strain harboring a plasmid (pCT105::aadA-2HA) was constructed with the *ct105::aadA* mutant allele and the sequence encoding 2HA fused to the 3′ end of *ct105*. HeLa cells were then infected for 40 h with *C*. *trachomatis* L2/434 harboring either pCT105-2HA or pCT105::aadA-2HA and analysed by immunoblotting. This confirmed that intron insertion within *ct105-2HA* prevented production of a protein detectable with anti-HA antibodies (Fig. 6A).

The intracellular growth of *C*. *trachomatis* L2/434 (parental strain), *ct105::aadA* mutant, and *ct105::aadA* mutant harboring CT105-2HA (complemented strain) was then monitored by quantifying the production of infectious progeny during the chlamydial developmental cycle. Based on this, no significant differences between the three strains were detected (Fig. 6B). However, when HeLa cells infected by the same strains for 24 h were fixed and analysed by immunofluorescence microscopy, this revealed that the inclusion size was smaller in the mutant and complemented strains by comparison to the parental strain (Fig. 6C,D). Because this difference in inclusion size between the mutant and the parental strain was also observed in the complemented strain, the defect is likely not due to the absence of CT105. Probably, intron insertion within *ct105* might have caused a polar effect in the neighbouring genes. In summary, this showed that CT105 is not required for *C*. *trachomatis* growth in tissue culture cells. In addition, considering the localization of CteG at the Golgi, we analysed if its morphology was altered in HeLa cells infected by the *ct105::aadA* mutant, by comparison to cells infected by the parental L2/434 strain. However, significant differences in Golgi morphology were not detected (Supplementary Fig. S11).

### CT105 induces a vacuolar protein sorting defect when ectopically expressed in *S*. *cerevisiae*

Given the localization of CT105 at the Golgi and plasma membrane in both infected and transfected mammalian cells, we hypothesized that the protein could interfere with eukaryotic vesicular trafficking. To test this, we asked if CT105 could cause a vacuolar protein sorting (VPS) defect in *S*. *cerevisiae*^37^, a eukaryotic model organism that has been often used to study bacterial effector proteins^38^. The VPS assay consists in a yeast reporter strain NSY01 (Supplementary Table S2) that produces a hybrid protein composed of carboxypeptidase Y and Invertase (CPY-Inv), which normally travels to the yeast vacuole but goes to the cell surface if trafficking is disrupted. As strain NSY01 does not produce endogenous Invertase, an enzyme that hydrolyzes sucrose into glucose and fructose, normal (Vps^+^) or aberrant (Vps^−^) trafficking of CPY-Inv can be scored by using an agar overlay solution indicating glucose production at the cell surface by formation of a brown precipitate (Vps^+^, white colonies; Vps^−^, brown colonies)^37^.

**Figure 7:**
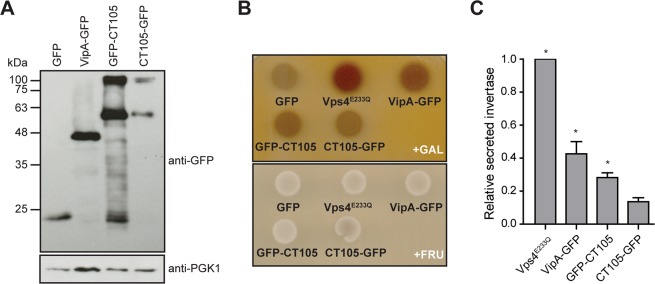
CT105 induces a vacuolar protein sorting defect in *S*. *cerevisiae*. *S*. *cerevisiae* reporter strain NSY01 producing CPY-invertase was transformed with plasmids encoding GFP (pKS84), VipA-GFP, GFP-CT105, or CT105-GFP, where their expression can be induced by galactose. A NSY01 derivative strain encoding a dominant-negative form of the yeast ATPase Vps4 (Vps4^E233Q^) was also used. Yeast strains are listed in Supplementary Table S2. (**A**) Whole cell extracts of *S*. *cerevisiae* NSY01 producing the indicated proteins were analyzed by immunoblotting with antibodies against GFP and PGK1 (yeast loading control), using SuperSignal West Pico detection kit (Thermo Fisher Scientific). (**B**) *S*. *cerevisiae* NSY01 strains encoding the indicated proteins were grown in solid medium in the presence of galactose (inducing conditions, +GAL) or in the presence of fructose (non-inducing conditions; +FRU). The vacuolar protein sorting (VPS) phenotype was analyzed using a sucrose overlay to assess activity of secreted invertase. A vacuolar protein sorting defect (VPS^−^) leads to the formation of a brown precipitate. (**C**) *S*. *cerevisiae* NSY01 strains encoding the indicated proteins were grown in liquid medium in the presence of galactose (inducing conditions) and the relative activity of secreted invertase was quantified (see Experimental Procedures). Data are mean ± standard error of the mean of five independent experiments. P-values were obtained by one-way ANOVA and Dunnett post-test analyses relative to GFP (0% of relative secreted invertase; not shown); *statistical significant (P < 0.05); ns, not significant.

Strain NSY01 was transformed with yeast expression plasmids encoding either CT105 fused to the N-terminus or C-terminus of GFP (CT105-GFP or GFP-CT105, respectively) under the control of a galactose inducible promoter (Fig. 7A). The first controls used were a NSY01 derivative strain that expressed only GFP (Fig. 7A), which leads to a Vps^+^ phenotype^39^, and NSY01 derivative strains expressing a dominant-negative form of the yeast ATPase Vps4 (Vps4^E233Q^)^40^ or the *Legionella pneumophila* effector protein VipA (Fig. 7A), both known to cause a Vps^−^ phenotype^37,39^. In the qualitative colorimetric enzymatic assay in solid media, it was consistently observed that GFP-CT105 or CT105-GFP induced a Vps^−^ phenotype (Fig. 7B). In addition, quantitative analyses in liquid media were performed and the amounts of secreted and total invertase were assessed for each strain. In this assay, both GFP-CT105 and CT105-GFP caused an increase in the levels of secreted invertase (relative to those of the control strain expressing GFP), but the difference was only statistically significant for GFP-CT105 (Fig. 7C). The C-terminal half of CT105 (amino acid residues 351 to 656), but not its N-terminal part (amino acid residues 1-443), was sufficient to induce a VPS defect in *S*. *cerevisiae* (Supplementary Fig. S12), indicating that the VPS-inhibitory activity of CT105 depends on its C-terminal region. Moreover, production in yeast of a fusion to GFP of another *C*. *trachomatis* type III secreted protein (CT142^27^) did not induce a VPS defect (Supplementary Fig. S12). Finally, production of GFP-CT105 or CT105-GFP caused no toxicity in *S*. *cerevisiae* NSY01 (Supplementary Fig. S12), indicating that the induction of the VPS defect by CT105 was not a consequence of an overall impact on yeast physiology. Thus, ectopic expression of CT105 in *S*. *cerevisiae* disrupted vesicular trafficking to the yeast vacuole.

## Discussion

We found that the *C*. *trachomatis* CT105 protein is delivered into the cytoplasm of infected host cells. At distinct times of the chlamydial developmental cycle, CT105 accumulates at the Golgi complex (16–30 h p.i.) and/or at the host cell plasma membrane (30–40 h p.i.). Because CT105 can be secreted by the T3S system of *Y*. *enterocolitica*^27^ and can interfere with vesicular trafficking in *S*. *cerevisiae* (Figs 7 and S12), this indicates that CT105 is a newly identified *C*. *trachomatis* T3S effector. However, thus far, we have no direct evidence for the ability of CT105 to interfere with vesicular trafficking in infected human cells and this must be analyzed directly in future studies. Overall, this work expands the portfolio of known chlamydial effector proteins and reveals the first *Chlamydia* protein that associates with the Golgi complex. Moreover, it illustrates that a single *C*. *trachomatis* effector can have distinct subcellular localizations, and possibly different functions, during the chlamydial developmental cycle. Such dual localization during infection as a likely means to diversify effector function is evocative of the *Salmonella* SopB effector, which initially localizes at the host cell membrane to mediate bacterial invasion but is then redirected to the *Salmonella*-containing vacuole to modulate bacterial intracellular growth^41^.

In addition to CT105, the *C*. *trachomatis* effector CT867, a deubiquitinase enzyme known as ChlaDub2^16^ or Cdu2^17^, has been detected at the inclusion membrane and at the host cell plasma membrane at distinct times of infection^22^. Such localization of CT105 and CT867/ChlaDub2/Cdu2 later in infection might indicate a function in host cell exit. It is unknown how CT105 and CT867/ChlaDub2/Cdu2 are differentially directed to different host cell localizations. In the case of CT105, it is unlikely that vesicular trafficking is involved as disruption of microfilaments and microtubules did not affect its localization at the plasma membrane in infected cells. We envision that host cytosolic CT105 is specifically directed to different subcellular localizations by covalent modifications it might be subjected to and/or by changes in the lipid and/or protein composition of host cellular membranes.

Ectopic expression of CT105 in yeast inhibited protein transport to the vacuole. While it remains to be directly tested, this observation suggested a potential capacity of CT105 to interfere with vesicular trafficking in infected cells. An appealing possibility for the function of CT105 would be that it contributes to nutrient acquisition by the inclusion and/or avoidance of fusion with hydrolytic compartments. However, the *C*. *trachomatis ct105::aadA* mutant did not reveal an obvious intracellular growth defect, which is not uncommon amongst *C*. *trachomatis* effector gene mutants characterized thus far^11,42,43^ and might reflect redundancy in effector function or inexistence of adequate infection models^7^. Furthermore, a putative activity of CT105 on vesicular trafficking in infected cells could also affect cytokine secretion^44^ or immune signaling^45^, which would not have an impact on chlamydial intracellular growth in tissue culture cells. Although the *ct105* mutant revealed smaller inclusions relative to the parental strain (suggesting a possible defect in vacuolar membrane expansion), this could not be restored by reintroduction of *ct105* in *trans*. The exact reasons for this need to be further analyzed, but the inability to complement intracellular growth-like phenotypes in intron insertional mutants of *C*. *trachomatis* was also recently described for *ct101/mrcA*^46^, while other specific phenotypes of this mutant strain could be complemented^46^.

The precise spatial and temporal subcellular targeting of bacterial effector proteins during infection is an essential feature of their function^47^. In other bacterial pathogens at least three other effector proteins have been shown to localize at the Golgi in infected host cells. These are EspI/NleA from *Escherichia coli*^48^, GobX from *L*. *pneumophila*^49^, and SteD from *Salmonella enterica*^50^. EspI/NleA interferes with coatomer protein II complex function, inhibits host cell protein secretion^51^, and disrupts intestinal tight junctions^52^. In addition, EspI/NleA inhibits NLRP3 inflammasome activation^53^. GobX displays E3 ubiquitin ligase activity, but how this promotes *L*. *pneumophila* infection is unknown^49^. Finally, SteD interferes with the activity of a host E3 ubiquitin ligase, leading to a reduction of surface-localized major histocompatibility complex class II molecules and suppression of T cell activation^50^. This illustrates that no specific function can be predicted for CT105 based on its localization at the Golgi.

The first 100 amino acid residues of CT105 are sufficient for Golgi localization when the protein is ectopically expressed in Hela cells. However, this region does not display obvious motifs found in other relevant eukaryotic or bacterial proteins, and the exact molecular determinants of the Golgi localization of CT105 remain to be identified. Other relevant questions are whether the same determinants are responsible for Golgi localization in infected cells and which characteristics of the full-length CT105 protein enable its targeting to the plasma membrane. Other effector proteins that localize at the Golgi in infected cells use distinct mechanisms directing them to the organelle^49,50,54,55^. In the case of EspI/NleA, Golgi targeting is partially mediated by a Postsynaptic Density95/Disc Large/Zonula Occludens-1 (PDZ) domain on its C-terminus^54^. CT105 also possesses a putative PDZ domain^56^, but the relevant residues are not involved in its subcellular localization after ectopic expression in mammalian cells (data not shown).

CT105 displays a striking variability within *C*. *trachomatis* serovars, revealing LGV-specific genetic and transcriptomic traits. This suggests that CT105 could have a specific function related with the unique characteristics of infections by these strains, which, unlike ocular and urogenital *C*. *trachomatis* strains, can infect mononuclear phagocytes and disseminate into regional lymph nodes^29,57^. It also indicates that features of the infection by LGV strains should be an evolutionary pressure to maintain an active *ct105* gene. Furthermore, among *Chlamydia* species, full-length orthologues of CT105 are only present in *C*. *muridarum* and *C*. *suis*, consistent with the fact that these species are the closer relatives of *C*. *trachomatis*^3^.

In summary, we identified CT105 as a T3S effector of *C*. *trachomatis* that localizes to the Golgi and plasma membrane in infected cells, and which can disrupt trafficking to the *S*. *cerevisiae* vacuole. Because CT105 is the first *Chlamydia* effector shown to associate with the Golgi complex, we propose to name it CteG (for *C*. *trachomatis* effector associated with the Golgi). The identification of CteG also leaves many questions to be addressed related to its function, subcellular targeting mechanisms, diversity, and specificity within *C*. *trachomatis* and among *Chlamydia* species.

## Materials and Methods

### Plasmids, primers, and DNA manipulation

The plasmids used in this work, their main characteristics and construction details, are described in Supplementary Table S3. The DNA primers used in their construction are listed in Supplementary Table S4. Plasmids were constructed and purified using standard molecular biology procedures. The accuracy of the nucleotide sequence of all the inserts in the constructed plasmids was confirmed by DNA sequencing. The backbone plasmids used in this work were: pSVP247^19^, a derivative of p2TK2-SW2^58^, was used to generate *C*. *trachomatis* expression plasmids encoding proteins with a 2HA at their C-termini; pmEGFP-N1 and pmEGFP-C1 [both constructed from pEGFP-N1 (Clontech) and pEGFP-C1 (Clontech), respectively, where the gene encoding EGFP was replaced by a gene encoding mEGFP from pLAMP1-mGFP^35^], and pEF6/*Myc*-His A (Thermo Fisher Scientific), were used to generate mammalian transfection plasmids; pYES2-GFP^59^ and pKS84^60^, were used to generate yeast expression plasmids encoding fusions to the C- or N-terminus of GFP, respectively. Furthermore, pBOMB4-Tet-mCherry^61^ and Lyn11-FRB-mCherry^62^ were used to amplify the P_*tet*_ and the nucleotide sequence encoding the Lyn11 peptide, respectively.

### Cells lines and transient transfection

HeLa 229 and Vero cells (from the European Collection of Cell Culture; ECACC) were maintained in high-glucose Dulbecco’s modified Eagle Medium (DMEM; Thermo Fisher Scientific) supplemented with heat-inactivated 10% (v/v) fetal bovine serum (FBS; Thermo Fisher Scientific) at 37 °C in a humidified atmosphere of 5% (v/v) CO_2_. Cells were checked for *Mycoplasma* by conventional PCR either using the Venor GeM Advance kit (Minerva Biolabs) or as described^63^. HeLa cells were transfected by using the jetPEI reagent (Polyplus-Transfection) according to the instructions of the manufacturer.

### Bacterial strains and growth conditions

*E*. *coli* TOP10 (Thermo Fisher Scientific) or NEB 10β (New England Biolabs) were used for construction and purification of plasmids, and *E*. *coli* ER2925 (New England Biolabs) was used to amplify and purify plasmids for transformation of C. *trachomatis*. *E*. *coli* strains were routinely grown in liquid or solid lysogeny broth (LB) medium (NZYTech) with the appropriate antibiotics and supplements at 37 °C.

*C*. *trachomatis* prototype strains B/Har36, C/TW3, E/Bour, L2/434, and L3/404 (from ATCC) and clinical strains F/CS465-95 and L2b/CS19-08 (from the collection of the Portuguese National Institute of Health) were propagated in HeLa 229 cells using standard techniques^64^. Transformation of *C*. *trachomatis* was done essentially as described by Agaisse and Derré^58^. The optimal antibiotic concentrations to select transformants were 1 U/ml of penicillin G, or 250 µg/ml of spectinomycin. Once established, the transformed strains were cultured in the presence of 10 U/ml of penicillin, or 500 µg/ml of spectinomycin, and plaque purified using Vero cells, as described^65^. Infection of mammalian cells by *C*. *trachomatis* and quantification of infection progeny was done as previously described^19^. Infected cells were harvested at the indicated times post-infection and analyzed by immunoblotting or immunofluorescence microscopy.

### Construction of a *C*. *trachomatis ct105::aadA* mutant strain

A *C*. *trachomatis ct105::aadA* mutant was generated using group II intron-based insertional mutagenesis, as previously described^66,67^. Briefly, intron-insertion sites in the *C*. *trachomatis* L2/434 *ctl0360* gene (orthologue of *ct105* in *C*. *trachomatis* strain D/UW3) were identified using the TargeTron algorithm (Sigma). Then, the intron in pDFTT3*aadA*^67^ (Supplementary Table S2) was retargeted for *ctl0360* using standard molecular biology procedures and the DNA primers listed in Supplementary Table S4. The *ctl0360* mutator plasmid (pDFTT296; Supplementary Table S3) was then used to transform *C*. *trachomatis* L2/434.

### Yeast strains and invertase assays

*S*. *cerevisiae* strains (Supplementary Table S2) used in this work were grown as previously described^37,39^. For the invertase assays, *S*. *cerevisiae* expressing GFP fusion proteins were grown in plates with yeast nitrogen base uracil dropout (YNB-Ura) supplemented with 2% (w/v) fructose for 3 days, at 25 °C. Cells were then streaked on YNB-Ura with 2% (w/v) fructose (non-inducing media) or 2% (w/v) galactose (inducing media) and grown for 3 days at 25 °C. Assays for qualitative and quantitative detection of invertase enzymatic activity were performed as described^37,68^. Relative secreted invertase was normalized by setting GFP as the minimum value and Vps4^E233Q^ as the maximum value.

### Antibodies, fluorescent dyes, and drug treatments

The list of antibodies and fluorescent dyes used is described in Supplementary Materials and Methods. To induce Golgi fragmentation, HeLa 229 cells were incubated with 1 µg/ml of BFA (Sigma; stock solution at 5 mg/ml in DMSO) for 1 h and then either fixed immediately or washed with medium without BFA and incubated for 1 h before fixation. To disrupt microtubules, HeLa 229 cells were incubated in the presence of 1 µg/ml nocodazole (stock solution at 5 mg/ml in DMSO; Sigma). To disrupt the actin cytoskeleton, *C*. *trachomatis*-infected cells were incubated in the presence of 2 µM cytochalasin D (stock solution at 5 mg/ml in DMSO; Sigma) or 500 nM latrunculin B (stock solution at 1 mg/ml in DMSO; Sigma) in serum-free DMEM.

### Immunoblotting

To harvest infected or transfected HeLa cells, they were washed once with phosphate-buffered saline (PBS) and then detached with TrypLE Express (Thermo Fisher Scientific) by incubation during 5 min at 37 °C in a 5% [v/v] CO_2_ atmosphere. The cells were then collected, pelleted by a brief centrifugation, washed 2 times with ice-cold PBS, and stored as a pellet at −20 °C until use. Prior to SDS-PAGE, the cells were thawed and resuspended in an appropriate volume of SDS-PAGE loading buffer. The proteins were further denatured by an incubation of 5 min at 100 °C, followed by addition of benzonase (Novagen) to destroy DNA and reduce the viscosity of the samples.

To prepare *Chlamydia*-enriched extracts, the infected HeLa cells were lysed by osmotic shock (15 min in sterile H_2_O). Lysates were centrifuged at 170 × *g* for 10 min at 4 °C. The supernatants were then centrifuged at 24,000 × *g* for 10 min at 4 °C and resulting pellets washed 2 times with ice-cold PBS. The bacteria-enriched pellets were resuspended in an appropriate volume of SDS-PAGE loading buffer and the proteins were further denatured by an incubation of 5 min at 100 °C.

To prepare yeast extracts, cells were grown for 3 days at 30 °C in YNB-Ura plates supplemented with 2% (w/v) fructose and then streaked into YNB-Ura supplemented with 2% (w/v) galactose. Cells corresponding to an optical density at 600 nm (OD_600_) of 2.5 were used for immunoblotting.

Samples were separated by 12% (v/v) SDS-PAGE and transferred onto 0.2 μm nitrocellulose membranes (Bio-Rad) using Trans-Blot Turbo Transfer System (BioRad). Immunoblot detection was done with SuperSignal West Pico Chemiluminescent Substrate (Thermo Fisher Scientific) or SuperSignal West Femto Maximum Sensitivity Substrate (Thermo Fisher Scientific) (as indicated in figure legends) and exposure to Amersham Hyperfilm ECL (GE Healthcare).

### Immunofluorescence microscopy

Infected HeLa cells were fixed either in PBS containing 4% (w/v) paraformaldehyde (PFA) for 10 min at room temperature or in methanol (−20 °C) for 10 min, as indicated in figure legends. For immunostaining, the antibodies were diluted in PBS containing 10% (v/v) horse serum (when fixation was done with PFA, 0.1% (v/v) Triton X-100 was added to allow permeabilization of cells). After immunolabelling, the cells were consecutively washed with PBS and H_2_O. The coverslips were assembled using Aqua-poly/Mount (Polysciences) on microscopy glass slides, and the cells were examined by conventional fluorescence microscopy or by confocal microscopy. Images were processed and assembled using Fiji software^69^. Analysis of Golgi redistribution around the *C*. *trachomatis* inclusion was performed as previously described^70^.

### RT-qPCR

To quantify the mRNA levels of *ct105* during the developmental cycle of *C*. *trachomatis* strains B/Har36, C/TW3, E/Bour, F/CS465-95, L2/434, L2b/CS19-08 and L3/404, RT-qPCR measurements were performed as previously described^71^, using primers listed in Supplementary Table S4. To compare by RT-qPCR the mRNA levels of *ct105* during the developmental cycle of *C*. *trachomatis* strains L2/434 or L2/434 harboring pCT105-2HA (pSVP264/pCT105-2HA; Supplementary Table S3), a 6-well plate seeded with HeLa 229 cells was inoculated by each strain at a multiplicity of infection of 50 and cells were harvested at the indicated times post infection by trypsinization. Total RNA was isolated using NZY Total RNA Isolation Kit (NZYtech). For each RNA sample, cDNA was generated using iScript cDNA Synthesis Kit (Bio-Rad) accordingly with the manufacturer’s instructions. RT-qPCR was performed using *ct105* and *16S* primers (Supplementary Table S4) and SsoFast EvaGreen Supermix (Bio-Rad). For each time point, quantitative PCR was performed on each cDNA sample (including an RT-negative sample) using Rotor Gene 6000 (Corbett Life Science). For normalization, ratios to the *16s* rRNA transcript were obtained. Data analysis was carried out with Rotor-Gene 6000 software. Unless otherwise indicated, RT-qPCR results were based in three independent experiments.

### Statistical analyses

Statistical analyses were done using GraphPad Prism, version 5.04 for Windows, GraphPad Software, San Diego California, USA (www.graphpad.com).

## Supplementary information

Supplementary Information

## Data Availability

All data generated or analyzed during this study are included in this published article (and its Supplementary Information files).
