# Supplementary material for: CteG is a Chlamydia trachomatis effector protein that associates with the Golgi complex of infected host cells
Source: Sci Rep. 2019 Apr 16;9:6133. doi: 10.1038/s41598-019-42647-3 (PMC6468002; doi:10.1038/s41598-019-42647-3)
Supplement: Supplementary file 1 — Supplementary Information [file 41598_2019_42647_MOESM1_ESM.pdf]

SUPPLEMENTARY INFORMATION

CteG is a *Chlamydia trachomatis* effector protein that associates with the Golgi complex of infected host cells

Sara V. Pais[1], Charlotte E. Key[2], Vítor Borges[3], Inês S. Pereira[1], João Paulo Gomes[3], Derek J. Fisher[2], and Luís Jaime Mota[1*]

[1]UCIBIO, Departamento de Ciências da Vida, Faculdade de Ciências e Tecnologia, Universidade Nova de Lisboa, Caparica, Portugal.

[2]Department of Microbiology, Southern Illinois University, Carbondale, Illinois, USA.

[3]Bioinformatics Unit, Department of Infectious Diseases, National Institute of Health, Lisbon, Portugal.

***Corresponding author:**

E-mail: ljmota@fct.unl.pt

SUPPLEMENTARY MATERIALS AND METHODS

Antibodies and fluorescents dyes

For immunoblotting the following primary antibodies were used: mouse monoclonal anti-chlamydial Hsp60 (A57-B9; Thermo Fisher Scientific; 1:1,000); goat polyclonal anti-MOMP of *C. trachomatis* (Abcam; 1:1,000 for immunoblotting); goat anti-*C. trachomatis* fluorescein isothiocyanate (FITC)-conjugated polyclonal antibody (Millipore; 1:100); rat monoclonal anti-HA (3F10; Roche; 1:1,000); mouse monoclonal anti-α-tubulin (clone B-5-1-2; Sigma Aldrich; 1:1,000); goat polyclonal anti-GFP (Sicgen; 1:1,000); mouse anti-PGK1 (Life Technologies; 1:1,000).

For immunofluorescence the following primary antibodies were used: goat polyclonal anti-major outer membrane protein (MOMP) of *C. trachomatis* (Abcam; 1:500), rat monoclonal anti-HA (3F10; Roche; 1:200); mouse anti-CT442[1] [a gift from Guangming Zhong; 1:200]; rabbit polyclonal anti-Cap1[2] [a gift from Agathe Subtil; 1:2,000]; rabbit polyclonal anti-GM130 (Sigma Aldrich; 1:200); mouse monoclonal anti-TGN46 (clone TGN46-8; Sigma Aldrich; 1:200).

For immunoblotting, the secondary antibodies were all horseradish peroxidase (HRP)-conjugated (GE Healthcare and Jackson ImmunoResearch; 1:10,000). For immunofluorescence, the secondary antibodies were all from Jackson ImmunoResearch and used at 1:200: Rhodamine Red-X-conjugated anti-rat; DyLight 488-conjugated anti-goat; AF488-conjugated anti-mouse; DyLight 405-conjugated anti-goat; AF488-conjugated anti-rabbit; Cy5-conjugated anti-mouse.

Acti-stain 670 phalloidin (Cytoskeleton) and 4′,6-diamidino-2-phenylindole (DAPI; Thermo Fisher Scientific) were used to stain filamentous actin and DNA, respectively.

SUPPLEMENTARY TABLE S1. Potential orthologues of *C. trachomatis* CT105 (CTL0360) in other *Chlamydiae*[a]

	Cover	Id	Protein	aa range (CTL0360)	aa range (target protein)	Protein length (aa)	E-value
C. abortus	38%	28%	CAB376	361-612	400-683	732	2e-14
C. avium	14%	30%	M832_01180	334-428	121-212	304	7e-4
	23%[b]	22%[b]	M832_01160	277-429	51-183	479	0.14[b]
C. caviae	41%	27%	CCA_00389	297-570	339-570	726	4e-20
	56%	26%	CCA_00390	282-651	416-782	898	3e-17
	12%	27%	CCA_00297	290-370	55-125	445	0.003
	15%	25%	CCA_00298	294-397	77-168	533	0.003
C. felis	69%	26%	CF0619	143-601	223-644	737	2e-19
	54%	24%	CF0618	301-656	406-758	816	2e-12
	15%	25%	CF0705	294-397	79-170	536	2e-05
	20%	23%	CF0706	290-422	55-176	447	9e-04
C. gallinacea	24%	22%	M787_003335	269-429	51-194	439	0.005
	26%[b]	24%[b]	M787_003340	300-427	69-179	451	0.057[b]
C. muridarum	99%	53%	TC_0381	1-651	1-644	650	0.0
C. pecorum	56%	28%	CpecS_0642	284-654	143-509	597	6e-29
	55%	26%	CpecS_0639	290-652	181-533	566	2e-24
	39%	23%	CpecS_0640	331-587	190-444	463	3e-16
C. pneumoniae	17%	30%	CPn0405	233-350	132-245	258	8e-10
	23%	22%	CPn0404	401-555	12-165	339	7e-07
C. psittaci	71%	25%	CPSIT_0422	189-654	72-523	610	9e-26
	35%	27%	CPSIT_0421	366-597	403-643	733	2e-13
C. suis	99%	47%	Q499_0113	4-654	7-648	650	7e-170
	72%	28%	Q499_0114	177-651	141-603	607	3e-44

[a] Orthologues of the *C. trachomatis* protein CT105 in other *Chlamydiae* were searched by PSI-BLAST. An individual PSI-BLAST search was performed between CT105 (using the amino acid sequence of CTL0360, from *C. trachomatis* serovar L2 strain 434/Bu) and a representative strain from each *Chlamydia* spp. (*C. abortus* strain S26/3, *C. avium* strain 10DC88, *C. caviae* strain GPIC, *C. felis* Fe/C-56, *C. gallinacean* 08-1274/3, *C. muridarum* strain Nigg, *C. pecorum* VR-629, *C. pneumoniae* strain CWL029, *C. psittaci* strain 6BC, *C. suis* strain MD56). Another individual PSI-BLAST search was performed between each protein and members of the other *Chlamydiae* families, but no significant hits were found. Cover, indicates % of coverage, and Id indicates % of identity; aa, amino acid.

[b] E-value worse than threshold.

SUPPLEMENTARY TABLE S2. ***Saccharomyces cerevisiae*** **strains used in this work**

**Strains**	**Genotype**	**Source/Reference**
NSY01	BHY10 diploid a/α, CPY-Inv, inv^-, ura^-	[3]
SCIF00	NSY01 *Pgal-gfp* (pKS84)	[4]
SCIF01	NSY01 *Pgal-vipA-gfp* (pIF206)	[4]
SCNS00	NSY01 $Pgal\text{-}vps4^{E233Q}$	[3]
SCSVP01	NSY01 *Pgal-gfp-ct105* (pCM35)	This work.
SCSVP02	NSY01 *Pgal-ct105-gfp* (pSVP293)	This work.
SCSVP03	NSY01 *Pgal-gfp* (pYES2-GFP)	This work.
SCSVP04	NSY01 *Pgal-gfp-ct142* (pLJM1051)	This work.
SCLJM01	NSY01 $Pgal\text{-}gfp\text{-}ct105_{1\text{-}300}$ (pLJM1080)	This work.
SCLJM02	NSY01 $Pgal\text{-}gfp\text{-}ct105_{1\text{-}1329}$ (pLJM1081)	This work.
SCLJM03	NSY01 $Pgal\text{-}gfp\text{-}ct105_{1052\text{-}1970}$ (pLJM1082)	This work.
SCLJM04	NSY01 $Pgal\text{-}gfp\text{-}ct105_{301\text{-}1970}$ (pLJM1085)	This work.

SUPPLEMENTARY TABLE S3. Plasmids used in this work.

Plasmid	Description[a]	Source/Reference
pBOMB4-Tet-mCherry	*C. trachomatis* vector for expression of proteins, under the control of a tetracycline-inducible promotor (Amp[R]).	[5]
pSVP247	Derivative of p2TK2--SW2 [6] for expression of proteins with a C-terminal double HA (2HA) tag. Contains the terminator of the *incDEFG* operon of *C. trachomatis* L2/434 (Amp[R]).	[7]
pEGFP-N1	Transfection vector. Fusions at the N-terminus of EGFP under the control of the CMV promoter (Km[R]).	Clontech
pEGFP-C1	Transfection vector. Fusions at the N-terminus of EGFP under the control of the CMV promoter (Km[R]).	Clontech
pLAMP1-mGFP	Transfection vector encoding human LAMP1 fused to monomeric GFP (mGFP) (Km[R])	Addgene plasmid # 34831[8]
Lyn11-FRB-mcherry	Mammalian expression vector encoding a Lyn Src kinase N-terminal sequence (Lyn11) fused to FRB (fragment of mTOR that binds rapamycin) and to mCherry.	Addgene plasmid # 38004[9]
pDFTT3*aadA*	Group II intron donor plasmid.	[10]
pmEGFP-N1/pALT1	Mammalian transfection vector for expression of proteins fused to the N-terminus of mEGFP under the control of the *CMV* promoter (Km[R]). The DNA fragment encoding mEGFP was amplified by PCR from pLAMP1-mEGFP using primers 1851 and 1852. The resulting DNA	This work.

	product was digested with AgeI and NotI and ligated into those sites of pGFP-N1.	
pmEGFP-C1/pALT2	Mammalian transfection vector for expression of proteins fused to the C-terminus of mEGFP under the control of the *CMV* promoter (Km^R). The DNA fragment encoding mEGFP was amplified by PCR from pLAMP1-mEGFP using primers 1852 and 1853. The resulting DNA product was digested with XhoI and NotI and ligated into those sites of pGFP-C1.	This work.
pEF6/*Myc*-His A	Mammalian transfection vector for expression of proteins, under the control of the *EF-1α* promoter (Km^R).	Thermo Fisher Scientific
pYES2-GFP	Yeast vector for expression of proteins fused to the C-terminus of GFP, under the control of a galactose-inducible promoter (Amp^R).	[11]
pKS84	Yeast vector for expression of proteins fused to the N-terminus of GFP, under the control of a galactose-inducible promoter (Amp^R).	[12]
pSVP261/pCT053-2HA	Encodes CT053-2HA under the control of the predicted *ct053* promoter. A DNA fragment containing *ct053* and its endogenous promoter was amplified from *C. trachomatis* L2/434 chromosomal DNA using primers 1677 and 1678. The resulting DNA product was digested with KpnI and NotI and inserted into those sites of pSVP247 (Amp^R).	This work.
pSVP263/pCT082-2HA	Encodes CT082-2HA under the control of the predicted *ct082* promoter. A DNA fragment containing c*t082* and its endogenous promoter	This work.

	was amplified from *C. trachomatis* L2/434 chromosomal DNA using primers 1679 and 1549. The resulting DNA product was digested with KpnI and NotI and inserted into those sites of pSVP247 (Amp^R).	
pSVP264/pCT105-2HA	Encodes CT105-2HA under the control of the predicted *ct105* promoter. A DNA fragment containing c*t105* and its endogenous promoter was amplified from *C. trachomatis* L2/434 chromosomal DNA using primers 1680 and 1552. The resulting DNA product was digested with KpnI and NotI and inserted into those sites of pSVP247 (Amp^R).	This work.
pSVP265/pCT429-2HA	Encodes CT429-2HA under the control of the predicted *ct429* promoter. A DNA fragment containing c*t429* and its endogenous promoter was amplified from *C. trachomatis* L2/434 chromosomal DNA using primers 1681 and 1682. The resulting DNA product was digested with KpnI and NotI and inserted into those sites of pSVP247 (Amp^R).	This work.
pSVP267/pCT849-2HA	Encodes CT849-2HA under the control of the predicted *ct849* promoter. A DNA fragment containing c*t849* and its endogenous promoter was amplified from *C. trachomatis* L2/434 chromosomal DNA using primers 1686 and 1687. The resulting DNA product was digested with KpnI and NotI and inserted into those sites of pSVP247 (Amp^R).	This work.
pSVP292	Transfection vector encoding CT105-2HA. A DNA fragment encoding CT105-2HA was amplified from pSVP264 using primers 1780 and	This work.

	1783. The resulting DNA product was digested with KpnI and NotI and inserted into those sites of pEF6/*Myc*-His A (Amp^R).	
pSVP293	Yeast expression vector encoding CT105-EGFP under the control of a galactose-inducible promoter. A DNA fragment containing *ct105* was amplified from *C. trachomatis* L2/434 chromosomal DNA using primers 1784 and 1785. The resulting DNA product was inserted into pKS84 by homologous recombination in yeast (Amp^R).	This work.
pSVP294	Transfection vector encoding LYN11-mEGFP. A DNA fragment encoding Lyn11 was amplified from pLYN11-FRB-mCherry using primers 1824 and 1848. A DNA fragment encoding mEGFP was amplified from pLAMP-1-mEGFP using primers 1847 and 1851. The two coding regions were fused by overlapping PCR using primers 1858 and 1859. The resulting DNA product was digested with HindIII and NotI and inserted into those sites of pEGFP-N1 (Km^R).	This work.
pSVP302/pTet-CT105-2HA	Encodes CT105-2HA under the control of the tetracycline-inducible promoter (P_{tet}). A DNA fragment containing P_{tet} was amplified from pBOMB4-Tet-mCherry using primers 1803 and 1804. A DNA fragment containing *ct105* was amplified from *C. trachomatis* L2/434 chromosomal DNA using primers 1805 and 1552. The two DNA fragments were then fused by overlapping PCR using primers 1803 and 1552. The resulting DNA product was digested with KpnI and NotI and inserted into those sites of pSVP247 (Amp^R).	This work.

pSVP303	Transfection vector encoding mEGFP-$CT105_{1-320}$. A DNA fragment encoding the first 320 amino acid residues of CT105 was amplified from *C. trachomatis* L2/434 chromosomal DNA using primers using primers 652 and 2129. The resulting DNA product was digested with KpnI and SalI and inserted into those sites of pmEGFP-C1 (Km^R).	This work.
pSVP310	Transfection vector encoding mEGFP-$CT105_{1-100}$. A DNA fragment encoding the first 100 amino acid residues of CT105 was amplified from *C. trachomatis* L2/434 chromosomal DNA using primers 652 and 2128. The resulting DNA product was digested with KpnI and SalI and inserted into those sites of pmEGFP-C1 (Km^R).	This work.
pSVP311	Transfection vector encoding mEGFP-$CT105_{\Delta 100-320}$. A DNA fragment encoding the first 100 amino acid residues of CT105 was amplified from *C. trachomatis* L2/434 chromosomal DNA using primers 652 and 2125. A DNA fragment encoding the region from the amino acid residue 320 to the C-terminus of CT105 was amplified from *C. trachomatis* L2/434 chromosomal DNA using primers 2124 and 653. The two DNA products were fused by overlapping PCR using primers 652 and 653. The resulting DNA product was digested with KpnI and SalI and inserted into those sites of pmEGFP-C1 (Km^R).	This work.
pSVP314	Transfection vector encoding mEGFP-$CT105_{\Delta 100}$. A DNA fragment encoding CT105 without its first 100 amino acid residues (but maintaining the ATG start codon) was amplified from *C. trachomatis* L2/434 chromosomal DNA	This work.

	using primers 2155 and 653. The resulting DNA product was digested with KpnI and SalI and inserted into those sites of pmEGFP-C1 (Km^R).	
pSVP316	Transfection vector encoding mEGFP-CT105$_{\Delta 320}$. A DNA fragment encoding CT105 without its first 320 amino acid residues (but maintaining the ATG start codon) was amplified from *C. trachomatis* L2/434 chromosomal DNA using primers 2161 and 653. The resulting DNA product was digested with KpnI and SalI and inserted into those sites of pmEGFP-C1 (Km^R).	This work.
pSVP319	Encodes CT105::aadA-2HA under the control of the predicted *ct105* promoter. A DNA fragment containing c*t105::aadA* and its endogenous promoter was amplified from *C. trachomatis* L2/434 *ct105::aadA* chromosomal DNA using primers 2254 and 1552. The resulting DNA product was digested with AgeI and NotI and inserted into those sites of pSVP247 (Amp^R).	This work.
pALT3	Transfection vector encoding CT105-mEGFP. A DNA fragment containing *ct105* was amplified from *C. trachomatis* L2/434 chromosomal DNA using primers 1778 and 1779. The resulting DNA product was digested with AgeI and SalI and inserted into those sites of pmEGFP-N1 (Km^R).	This work.
pALT4	Transfection vector encoding mEGFP-CT105. A DNA fragment containing *ct105* was amplified from *C. trachomatis* L2/434 chromosomal DNA using primers 652 and 653. The resulting DNA product was digested with KpnI and SalI and inserted into those sites of pmEGFP-C1 (Km^R).	This work.

pCM35	Yeast expression vector encoding GFP-CT105 under the control of a galactose-inducible promoter. A DNA fragment containing *ct105* was amplified from *C. trachomatis* L2/434 chromosomal DNA using primers 799 and 812. The resulting DNA product was digested with BglII and PstI, and with PstI and NotI. The two DNA fragments obtained were inserted into BamHI and NotI sites of pGFP-YES2 (Amp^R).	This work.
pDFTT296	Derivative of pDFTT3*aadA* carrying a group II intron targeting sequence of *ctl0360* from *C. trachomatis* strain L2/434 (orthologue of *ct105* in strain D/UW3). The 5' intron sequence was amplified by PCR using primers 1860, 1861, 1862, and 1922. The resulting ~350 bp DNA product was digested with HindIII and BsrG1 and ligated into those sites of pDFTT3*aadA* (Spc^R).	This work.
pLJM1051	Yeast expression vector encoding GFP-CT142 under the control of a galactose-inducible promoter. A DNA fragment containing *ct142* was amplified from *C. trachomatis* L2/434 chromosomal DNA using primers 813 and 814. The resulting DNA product was digested with BamHI and EcoRI and inserted into those sites of pGFP-YES2 (Amp^R).	This work.
pLJM1080	Yeast expression vector encoding GFP-$CT105_{1-100}$ under the control of a galactose-inducible promoter. A DNA fragment containing $ct105_{1-300}$ was amplified from *C. trachomatis* L2/434 chromosomal DNA using primers 812 and 2458. The resulting DNA product was digested with BglII and NotI. The DNA fragment obtained was	This work.

	inserted into BamHI and NotI sites of pGFP-YES2 (Amp^R).	
pLJM1081	Yeast expression vector encoding GFP-$CT105_{1-443}$ under the control of a galactose-inducible promoter. A DNA fragment containing $\mathit{ct105}_{\mathit{1-1329}}$ was amplified from *C. trachomatis* L2/434 chromosomal DNA using primers 812 and 2462. The resulting DNA product was digested with BglII and NotI. The DNA fragment obtained was inserted into BamHI and NotI sites of pGFP-YES2 (Amp^R).	This work.
pLJM1082	Yeast expression vector encoding GFP-$CT105_{351-656}$ under the control of a galactose-inducible promoter. A DNA fragment containing $\mathit{ct105}_{\mathit{1052-1970}}$ was amplified from *C. trachomatis* L2/434 chromosomal DNA using primers 799 and 2463. The resulting DNA product was digested with BamHI and NotI and inserted into those sites of pGFP-YES2 (Amp^R).	This work.
pLJM1085	Yeast expression vector encoding GFP-$CT105_{101-656}$ under the control of a galactose-inducible promoter. A DNA fragment containing $\mathit{ct105}_{\mathit{301-1970}}$ was amplified from *C. trachomatis* L2/434 chromosomal DNA using primers 799 and 2459 after overlapping PCR with primers 2459 and 2461, and 2460 and 799 (silent mutation to remove BamHI site). The resulting DNA product was digested with BamHI and NotI and inserted into those sites of pGFP-YES2 (Amp^R).	This work.

[a]Amp^R: Ampicillin resistance; Km^R: Kanamycin resistance; Spc^R: Spectinomycin resistance.

SUPPLEMENTARY TABLE S4. DNA primers used in this work.

Number	Description	Sequence (5' - 3')	Restriction site
652	Forward primer to construct pALT4, pSVP310, pSVP303, pSVP311	GATCGATCGTCGACTCATTTGGTATTGGTAGTGC	SalI
653	Reverse primer to construct pALT4, pSVP314, pSVP316, pSVP311	GATCGGTACCCTAGATAGAGGAGCTTTGCACACC	KpnI
799	Reverse primer to construct pCM35, pLJM1082, pLJM1085	ATAAGAATGCGGCCGCCTAGATAGAGGAGCTTTGCACACC	NotI
812	Forward primer to construct pCM35, pLJM1080, pLJM1081	GATCAGATCTTCATTTGGTATTGGTAGTGC	BglII
813	Forward primer to construct pLJM1051	GATCGGATCCAGTGATTCTGACAAAATTATTAATG	BamHI
814	Reverse primer to construct pLJM1051	GATCGAATTCTTATCCTCCTATCTCTGGG	EcoRI
1549	Reverse primer to construct pSVP263	GATCGCGGCCGCGTGAATCGCCGCCTGCATCCTC	NotI
1552	Reverse primer to construct pSVP264, pSVP302 and pSVP319	GATCGCGGCCGCGGATAGAGGAGCTTTGCACACC	NotI
1677	Forward primer to construct pSVP261	GATCGGTACCTTTCCATGAAGAATCCATCG	KpnI
1678	Reverse primer to construct pSVP261	GATCGCGGCCGCGCCATTCATTCGCGTCAGGATC	NotI
1679	Forward primer to construct pSVP263	GATCGGTACCGGTTAGGCATGAGTGCCTGC	KpnI

1680	Forward primer to construct pSVP264, pMBC5	GATCGGTACCTTCTTTATTATTGAGAAACG	KpnI
1681	Forward primer to construct pSVP265	GATCGGTACCTTATGAGCGAGGTTGTGAGC	KpnI
1682	Reverse primer to construct pSVP265	GATCGCGGCCGCGTGAACGGCTCTTCTTACGTCCACG	NotI
1686	Forward primer to construct pSVP267	GATCGGTACCTTCCAAAATCCTTTTTAGGG	KpnI
1687	Reverse primer to construct pSVP267	GATCGCGGCCGCGAGACAGGGGTTTATTTAATTGG	NotI
1778	Forward primer to construct pALT3	GATCGTCGACACCATGAGCTTTGGTATTGGTAGTGCTTG	SalI
1779	Reverse primer to construct pALT3	GATCACCGGTGGGATAGAGGAGCTTTGCACACCTGC	AgeI
1780	Forward primer to construct pSVP292	GATCGGTACCACCATGAGCTTTGGTATTGGTAGTGCTTG	KpnI
1783	Reverse primer to construct pSVP292	GATCGCGGCCGCTTAAGCATAATCAGGAACATCATACG	NotI
1784	Forward primer to construct pSVP293	GTTAATATACCTCTATACTTTAACGTCAAGGAGAAAAAAC ATGTCATTTGGTATTGGTAGTGC	-
1785	Reverse primer to construct pSVP293	GGATGGGCACCACTCCAGTGAAAAGTTCTTCTCCTTTACTGATAGAGGAGCTTTGCACACC	-
1799	Forward primer to construct pMBC2	GGAATTCCATATGGGCAGTGAGGGTAACAGCGAA GAAGG	NdeI
1800	Forward primer to construct pMBC3	GGAATTCCATATGTCTGGTGCTGCTTCTGCTGTAT GCC	NdeI

1801	Reverse overlap primer to construct pMBC1	CACCAGCGTTTCTGGGTGACCTGAGGCG GCGTCT GAACCCG	-
1802	Forward overlap primer to construct pMBC1	CGGGTTCAGACGCCGCCTCAGGTCACCC AGAAAC GCTGGTG	-
1803	Forward primer to construct pSVP302	GATCGGTACCTTAAGACCCACTTTCACAT TTAA	KpnI
1804	Reverse overlap primer to construct pSVP302	CTACCAATACCAAATGACATTTCACTTTTC TCTATCACTGATAGGGAGTGG	-
1805	Forward overlap primer to construct pSVP302	CCACTCCCTATCAGTGATAGAGAAAAGTG AAATGTCATTTGGTATTGGTAG	-
1813	Forward overlap primer to construct pMBC5	CCGGGAGTTAAAGGTATGGGCAGTGAGG GTAACA GCGAAGAAGG	-
1824	Forward primer to construct pSVP294	GATCAAGCTTACCATGGGATGTATAAAAT CAAAAGGGAAAGACAGCAGA	HindIII
1847	Forward overlap primer to construct pSVP294	TCAAAAGGGAAAGACAGCAGAGTGAGCA AGGGCGAGGAGCTG	-
1848	Reverse overlap primer to construct pSVP294	CAGCTCCTCGCCCTTGCTCACTCTGCTGT CTTTCCCTTTTGA	-
1851	Reverse primer to construct pmEGFP-N1 and pSVP294	GATCGCGGCCGCTTACTTGTACAGCTCGT CCATGCC	NotI
1852	Forward primer to construct pmEGFP-N1 and pmEGFP-C1	GATCACCGGTCGCCACCATGGTGAGCAA GGGCGAGCTG	AgeI
1853	Reverse primer to construct pmEGFP-C1	GATCCTCGAGACTTGTCAGCTCGTCCATG CC	XhoI

1858	Forward primer to construct pSVP294	GATCAAGCTTACCATGGGATG	HindIII
1859	Reverse primer to construct pSVP294	GATCGCGGCCGCTTACTTGTAC	NotI
1860	Group II intron retargeting (CT105_261|262s_IBS1/2)	AAAAAAGCTTATAATTATCCTTACATGGCGGACGCGTGCGCCCAGATAGGGTG	-
1861	Group II intron retargeting<br><br>(CT105_261|262s EBS1/delta)	CAGATTGTACAAATGTGGTGATAACAGATAAGTCGGACGCGGTAACTTACCTTTCTTTGT	-
1862	Group II intron retargeting (CT105_261|262s EBS2)	TGAACGCAAGTTTCTAATTTCGGTTCCATGTCGATAGAGGAAAGTGTCT	-
1922	Group II intron retargeting (EBS universal)	CGAAATTAGAAACTTGCGTTCAGTAAAC	-
1932	Forward primer for *16S* RT-qPCR	GCGAAGGCGCTTTTCTAATTTAT	-
1933	Reverse primer for *16S* RT-qPCR	CCAGGGTATCTAATCCTGTTTGCT	.
1934	Forward primer for *ct105* RT-qPCR	ATGGAGCCGTTTGTGTGGTT	-
1935	Reverse primer for *ct105* RT-qPCR	CCTTCTTCGCTGTTACCCTCACT	-
2124	Forward overlap primer to construct pSVP311	ACCAAAAATATTCTGCTAGTGGATATCCTTCTTGCGGATG	-
2125	Reverse overlap primer to construct pSVP311	CATCCGCAAGAAGGATATCCACTAGCAGAATATTTTTGGT	-

2128	Reverse primer to construct pSVP310	GATCGGTACCCTAACTAGCAGAATATTTTTGGTAGC	KpnI
2129	Reverse primer to construct pSVP303	GATCGGTACCCTAACACGCACTTGCGCAGCAAGGC	KpnI
2155	Forward primer to construct pSVP314	GATCGATCGTCGACGGAGTAAGTCTTACATCTATATCC	SalI
2161	Forward primer to construct pSVP316	GATCGATCGTCGACGGATATCCTTCTTGCGGATGTGC	SalI
2254	Forward primer to construct pSVP319	GATCACCGGTCCTTCTTTATTATTGAGAAACG	AgeI
2458	Reverse primer to construct pLJM1080	ATAAGAATGCGGCCGCCTAACTAGCAGAATATTTTTGGTAGC	NotI
2459	Forward primer to construct pLJM1085	GATCGGATCCGGAGTAAGTCTTACATCTATATCC	BamHI
2460	Overlapping forward primer to construct pLJM1085	GCCTCAAGGATCAGGATCTTCTGCAAGAGGTGCTGG	-
2461	Overlapping reverse primer to construct pLJM1085	CCAGCACCTCTTGCAGAAGATCCTGATCCTTGAGGC	-
2462	Reverse primer to construct pLJM1081	ATAAGAATGCGGCCGCCTAGTTATCTAAAATTCCTGCACAAG	NotI
2463	Forward primer to construct pLJM1082	GATCGGATCCCAAGAGTCTCCTGCAGAAGAAGC	BamHI

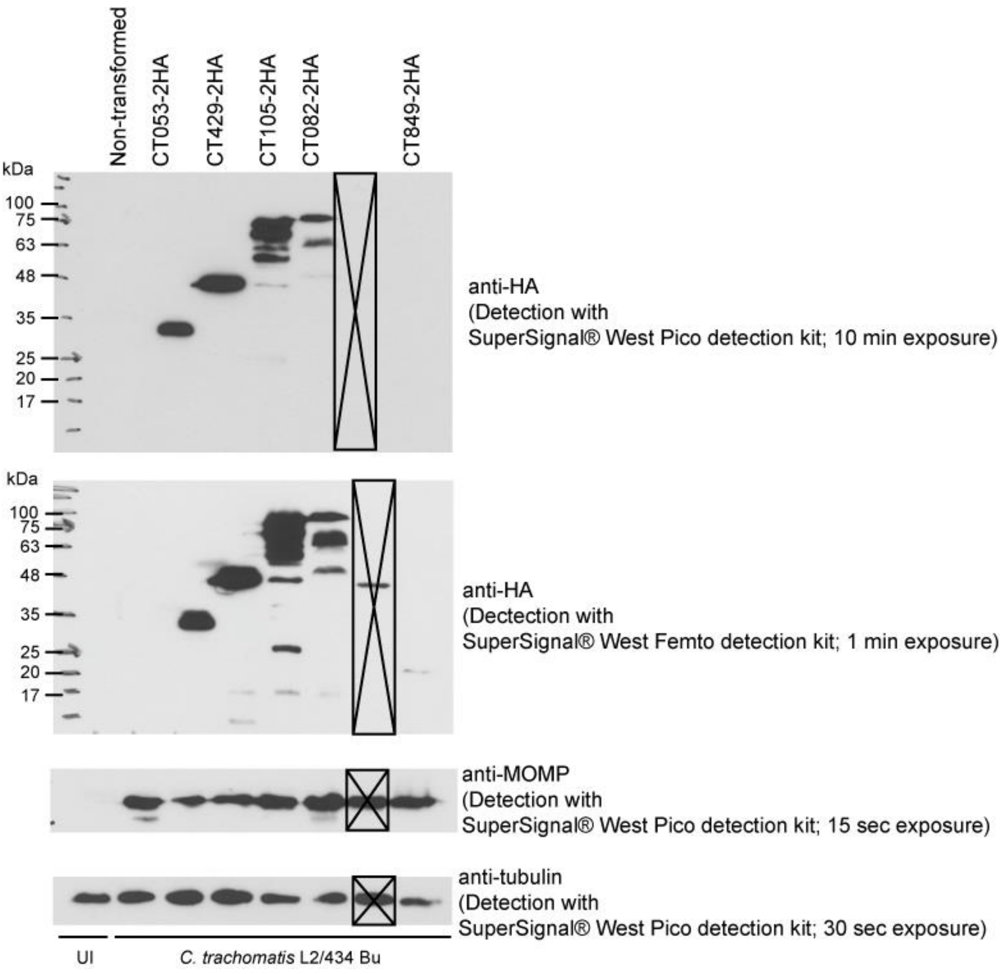

SUPPLEMENTARY FIGURE S1. Raw immunoblots of Fig. 1A

Unprocessed scans of immunoblots with antibodies against HA, *C. trachomatis* major outer membrane protein (MOMP) and tubulin indicating the detection kits and times of exposure used.

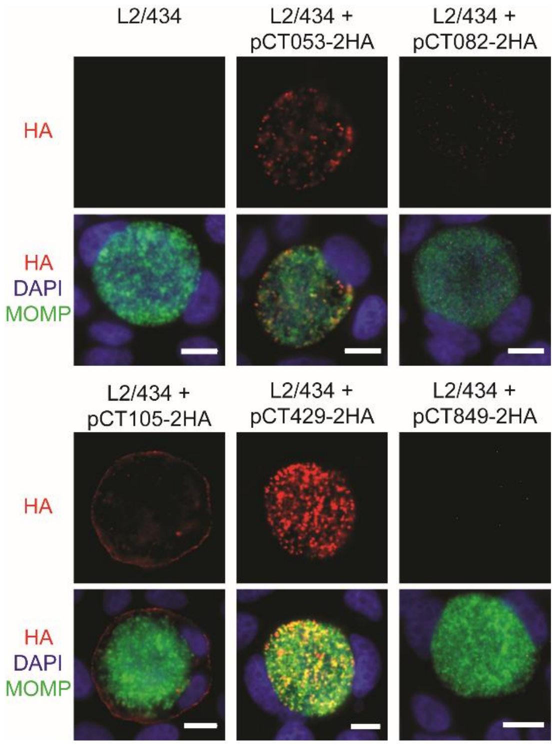

SUPPLEMENTARY FIGURE S2. Analysis of the production of candidate chlamydial type III secretion (T3S) effectors and of their delivery into the cytoplasm of *Chlamydia trachomatis*-infected cells.

HeLa cells were infected by *C. trachomatis* L2/434 or L2/434-derivative strains harboring plasmids encoding candidate T3S effectors (CT053, CT082, CT105, CT429, CT849) with a double hemagglutinin (2HA) tag at their C-termini. At 40 h post-infection, cells were fixed with 4% (w/v) paraformaldehyde, stained with 4',6-diamidino-2-phenylindole (DAPI; blue), immunolabeled with antibodies against HA (red) and *C. trachomatis* major outer membrane protein (MOMP; green), and appropriate fluorophore-conjugated secondary antibodies, and imaged by fluorescence microscopy. Scale bars, 10 µm.

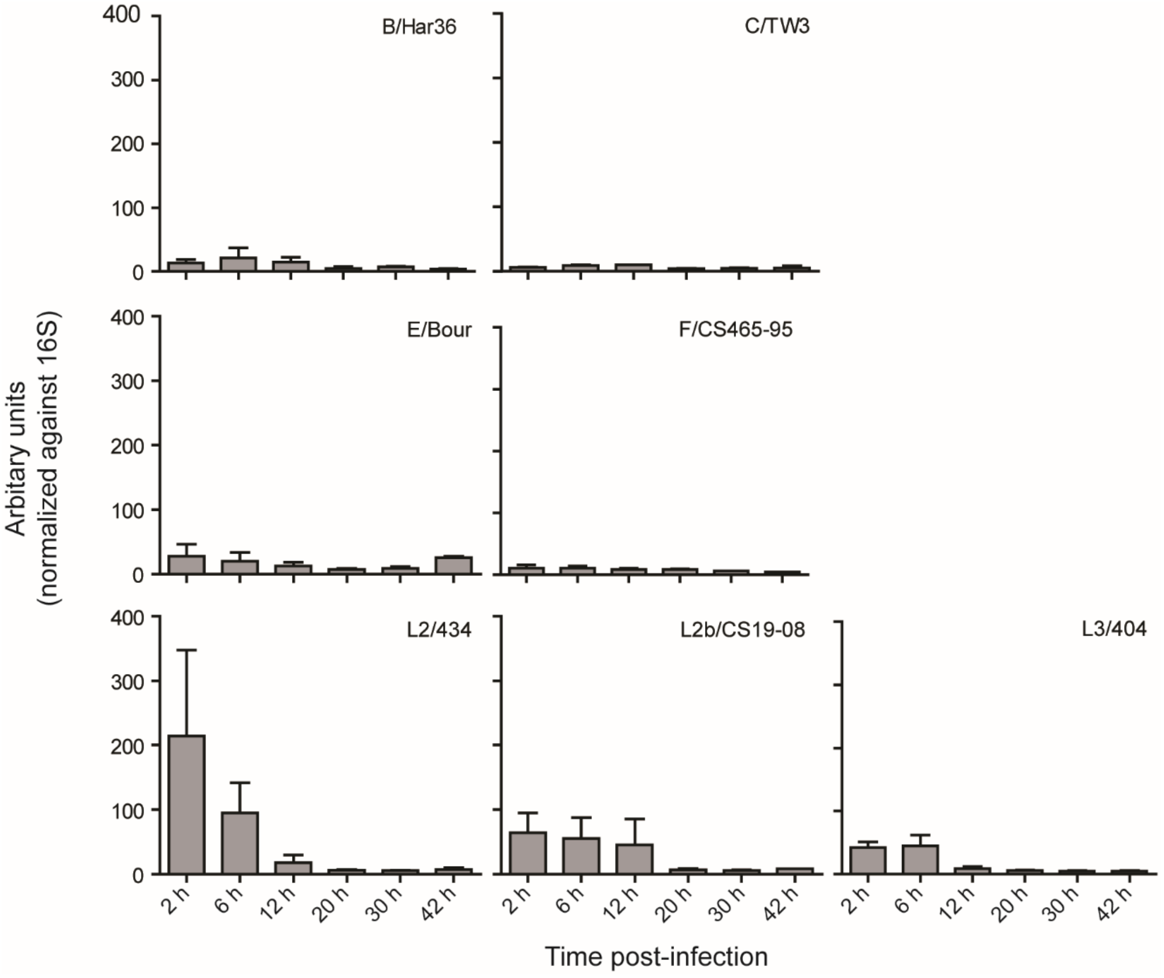

SUPPLEMENTARY FIGURE S3. mRNA levels of *ct105* in different *Chlamydia trachomatis* strains.

The mRNA levels of *ct105* were analyzed by real-time quantitative PCR (RT-qPCR) during the developmental cycle of *C. trachomatis* ocular (B/Har36, C/TW3), urogenital (E/Bour, F/CS465-95) or lymphogranuloma venereum (LGV) (L2/434, L2b/CS19-08, L3/404) strains, at the indicated time-points. The expression values (mean ± standard error of the mean) resulted from raw RT-qPCR data (x 10^5) of the *ct105* gene normalized to that of the *16S* rRNA gene and are from two independent experiments (except for strain C/TW3 where only one experiment was performed).

A

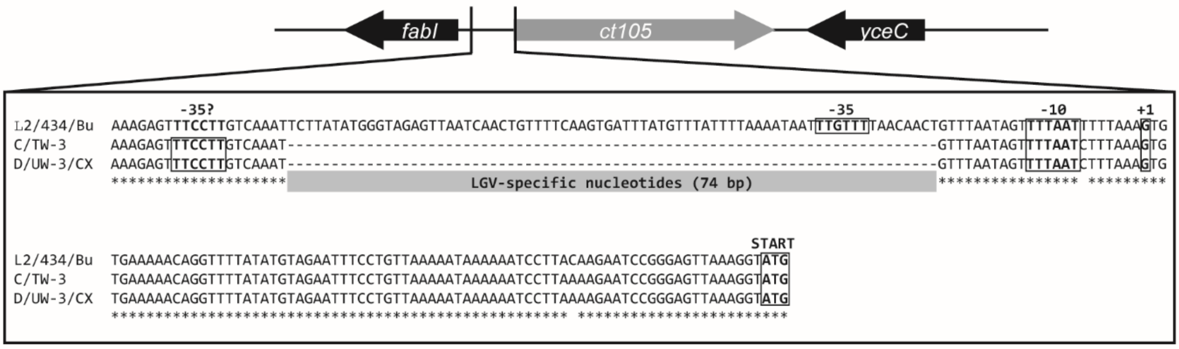

B

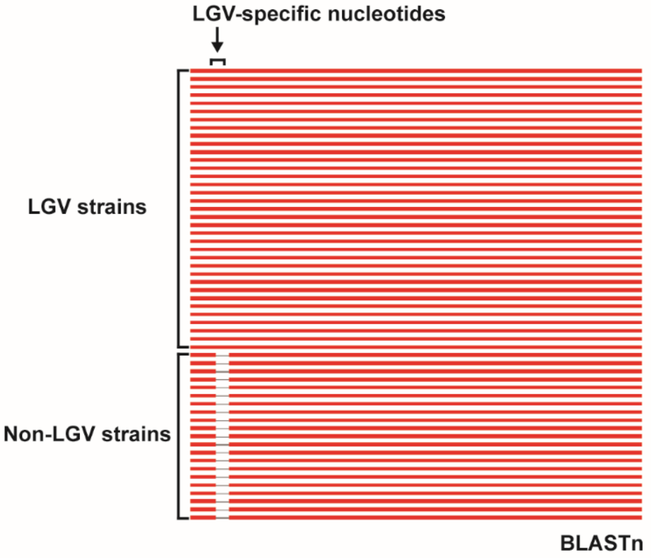

SUPPLEMENTARY FIGURE S4. Putative promoter region of *ctl0360/ct105* in different *Chlamydia trachomatis* strains.

A. Representation of the genetic organization of *ctl0360/ct105* and the nucleotide sequence of the putative promoter region of *ctl0360/ct105* in *C. trachomatis* L2/434 (LGV strain), C/TW-3 (ocular strain) and D/UW-3/CX (urogenital strain).The sequences have the annotation of the putative transcription start site (+1), the predicted -10 and -35 hexamers, the start codon (Start) and the 74-bp LGV-specific nucleotides, based on the transcription start site identified in *C. trachomatis* LGV strain L2b/UCH-1/proctitis (Albrecht et al., 2010).

B. BLAST nucleotide (BLASTn) analysis of the genomic region of *ctl0360/ct105* highlights the LGV-specific nucleotides (arrow).

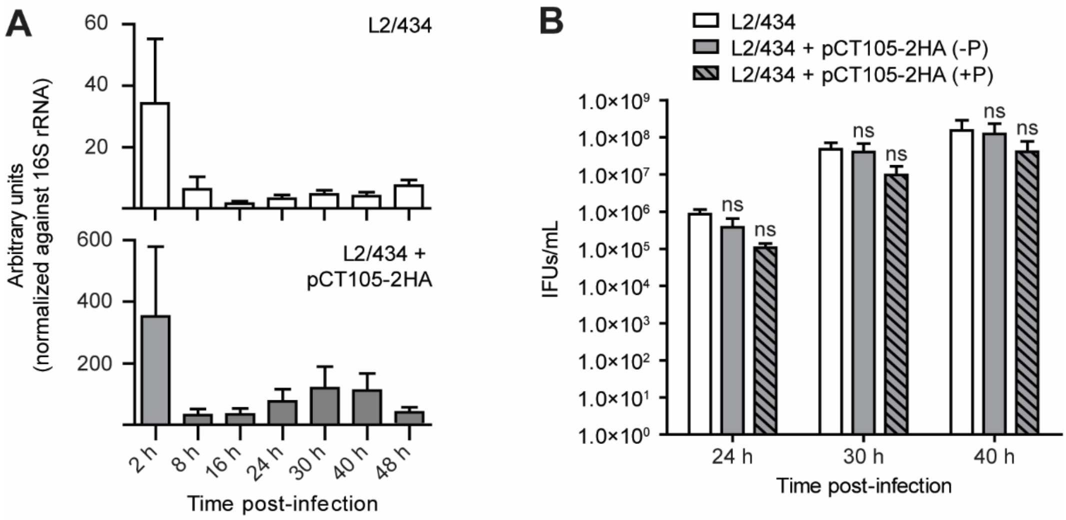

SUPPLEMENTARY FIGURE S5. Characterization of *Chlamydia trachomatis* L2/434 strain harboring pCT105-2HA.

A. *ct105* mRNA levels of *C. trachomatis* L2/434 and *C. trachomatis* L2/434 harboring pCT105-2HA (Supplementary Table S3) were analyzed by real-time quantitative PCR (RT-qPCR) at the indicated times post-infection. The expression values (mean ± standard error of the mean) resulted from raw RT-qPCR data (x 10^5) of the *ct105* gene normalized to that of the *16S* rRNA gene and are from three independent experiments.

B. HeLa cells were infected with the indicated *C. trachomatis* strains at a multiplicity of infection of 1, in the absence (-P) or presence (+P) of 1 U/mL of penicillin G. Recoverable inclusion forming units (IFUs) were determined at 24, 30, and 40 h post-infection. Data are mean ± standard error of the mean of three independent experiments. P-values were obtained by one-way ANOVA and Dunnett post test analyses relative to the parental L2/434 strain; ns, not significant ($P > 0.05$).

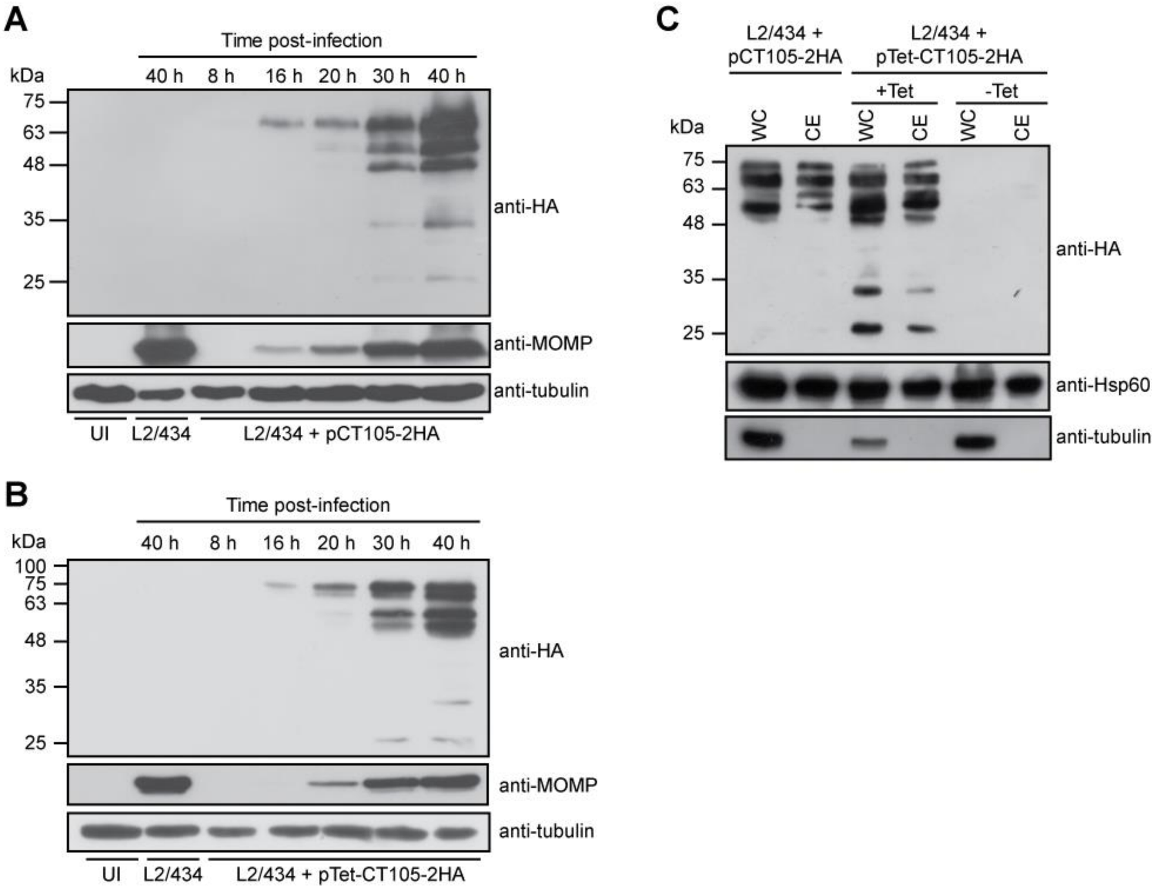

SUPPLEMENTARY FIGURE S6. Analyses of CT105-2HA production by immunoblotting.

HeLa cells were either left uninfected or infected, as indicated, by *Chlamydia trachomatis* L2/434, L2/434 harboring pCT105-2HA (a plasmid encoding CT105 with a double hemagglutinin tag at its C-terminus [CT105-2HA], expressed under the control of the *ct105* promoter; Supplementary Table S3), or L2/434 harboring pTet-CT105-2HA (a plasmid encoding CT105-2HA, expressed under the control of the tetracycline promoter; Supplementary Table S3).

A and **B.** Total extracts of uninfected (UI) cells or of cells infected by the indicated strains and at the indicated times were analyzed by immunoblotting with antibodies against HA, *C. trachomatis* major outer membrane protein (MOMP; bacterial loading control) and α-tubulin (HeLa loading control). In cells infected by L2/434 harboring pTet-CT105-2HA, anhydrotetracycline was added to 20 ng/mL at time zero of infection.

C. HeLa cells were infected for 40 h with the indicated strains. In cells infected by L2/434 harboring pTet-CT105-2HA, anhydrotetracycline was either not added (-Tet) or added (+Tet) to 20 ng/mL at time zero of infection. Whole cell (WC) and *Chlamydia*-enriched (CE) extracts (see Experimental Procedures) were analyzed by immunoblotting with antibodies against HA, *C. trachomatis* Hsp60 (bacterial loading control) and α-tubulin (HeLa loading control).

Immunoblotting detection was done using SuperSignal West Pico detection kit (Thermo Fisher Scientific) for MOMP, Hsp60 and α-tubulin, or SuperSignal West Femto detection kit (Thermo Fisher Scientific) for HA.

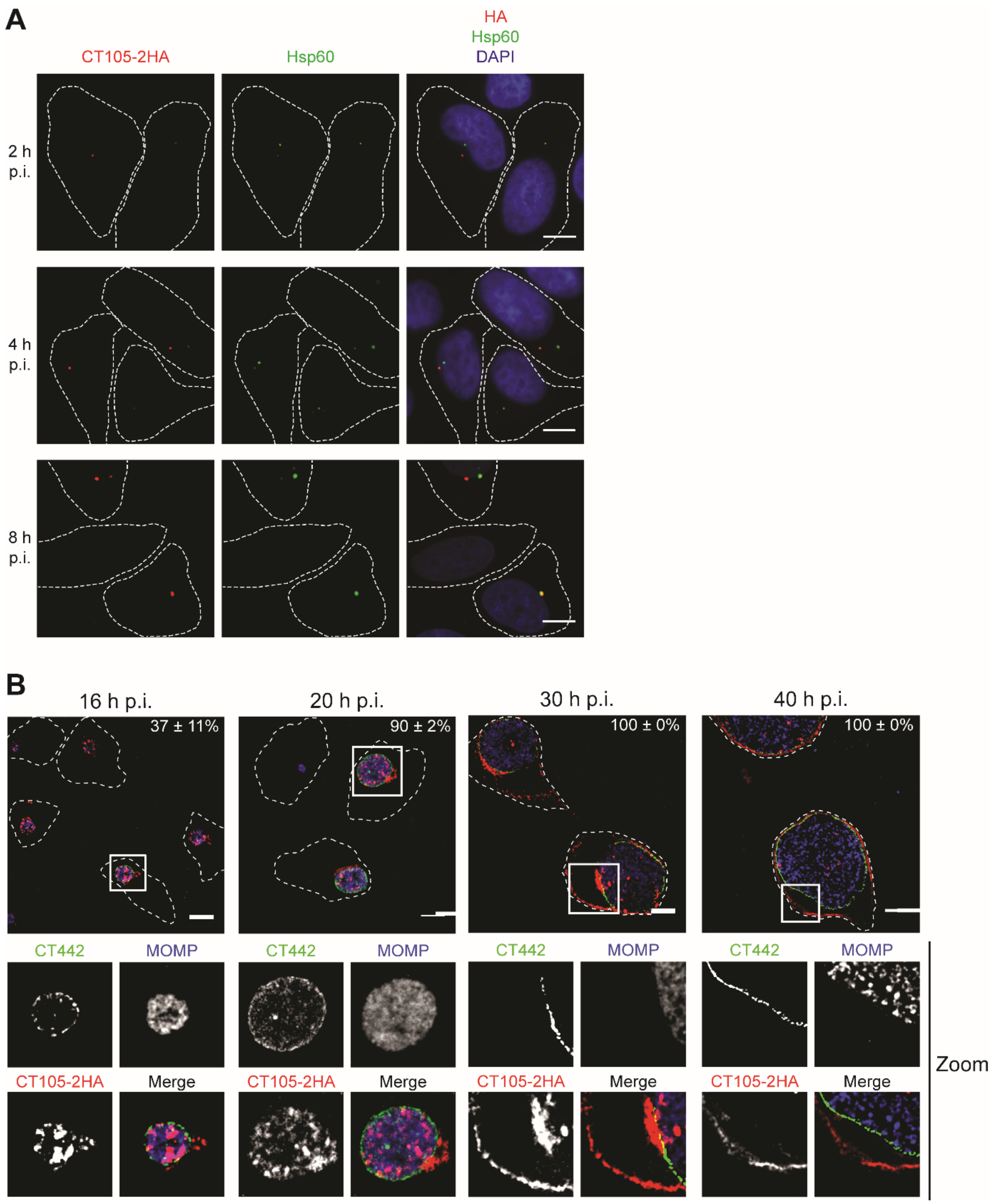

SUPPLEMENTARY FIGURE S7. Analyses of CT105-2HA production possible delivery into the cytoplasm of *Chlamydia trachomatis*-infected cells by immunofluorescence microscopy.

HeLa cells were infected by *C. trachomatis* L2/434 harboring pCT105-2HA (Supplementary Table S3) for 2, 4, 16, 20, 30, and 40 h post-infection (p.i.), and fixed with 4% (w/v) paraformaldehyde.

A. Cells were stained with 4',6-diamidino-2-phenylindole (DAPI; blue), immunolabeled with antibodies against HA (red) and *C. trachomatis* Hsp60 (green), and appropriate fluorophore-conjugated secondary antibodies, and imaged by fluorescence (2 h and 4 h p.i.) or confocal fluorescence (8 h p.i.) microscopy. Confocal image corresponds to single *z* section. Scale bars, 10 µm.

B. Cells were immunolabeled with antibodies against HA (red), C. trachomatis MOMP (blue), and the inclusion membrane protein CT442 (green), and appropriate fluorophore-conjugated secondary antibodies, and imaged by confocal fluorescence microscopy. Images correspond to single z sections. The values indicate the number of infected cells showing CT105-2HA outside of the inclusion, in the cytoplasm of the infected host cell. In the area delimited by a white square (upper panels) images were zoomed (lower panels). Data are the mean ± standard error of the mean of three independent experiments (n ≥ 25). Scale bars, 10 µm.

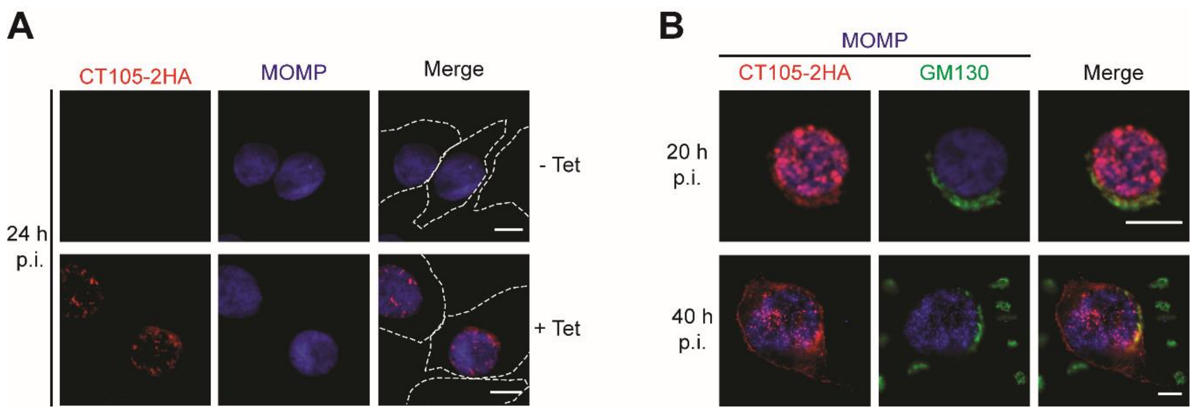

SUPPLEMENTARY FIGURE S8. Subcellular localization in infected cells of bacterially-delivered CT105-2HA expressed in *C. trachomatis* under the control of the tetracycline promoter.

HeLa cells were infected by *C. trachomatis* L2/434 harboring pTet-CT105-2HA (Supplementary Table S3) and fixed with 4% (w/v) paraformaldehyde at the indicated times post-infection (p.i.).

A. During infection, anhydrotetracycline was either not added (-Tet) or added (+Tet) to 20 ng/ml at time zero of infection. Fixed cells were immunolabeled with antibodies against HA (red) and *C. trachomatis* major outer membrane protein (MOMP; blue), and appropriate fluorophore-conjugated secondary antibodies.

B. During infection, anhydrotetracycline was added to 20 ng/mL at time zero of infection. Fixed cells were immunolabeled with antibodies against HA (red), *C. trachomatis* MOMP (blue), and GM130 (green), followed by appropriate fluorophore-conjugated secondary antibodies.

Cells were imaged by fluorescence microscopy. Scale bars, 10 µm.

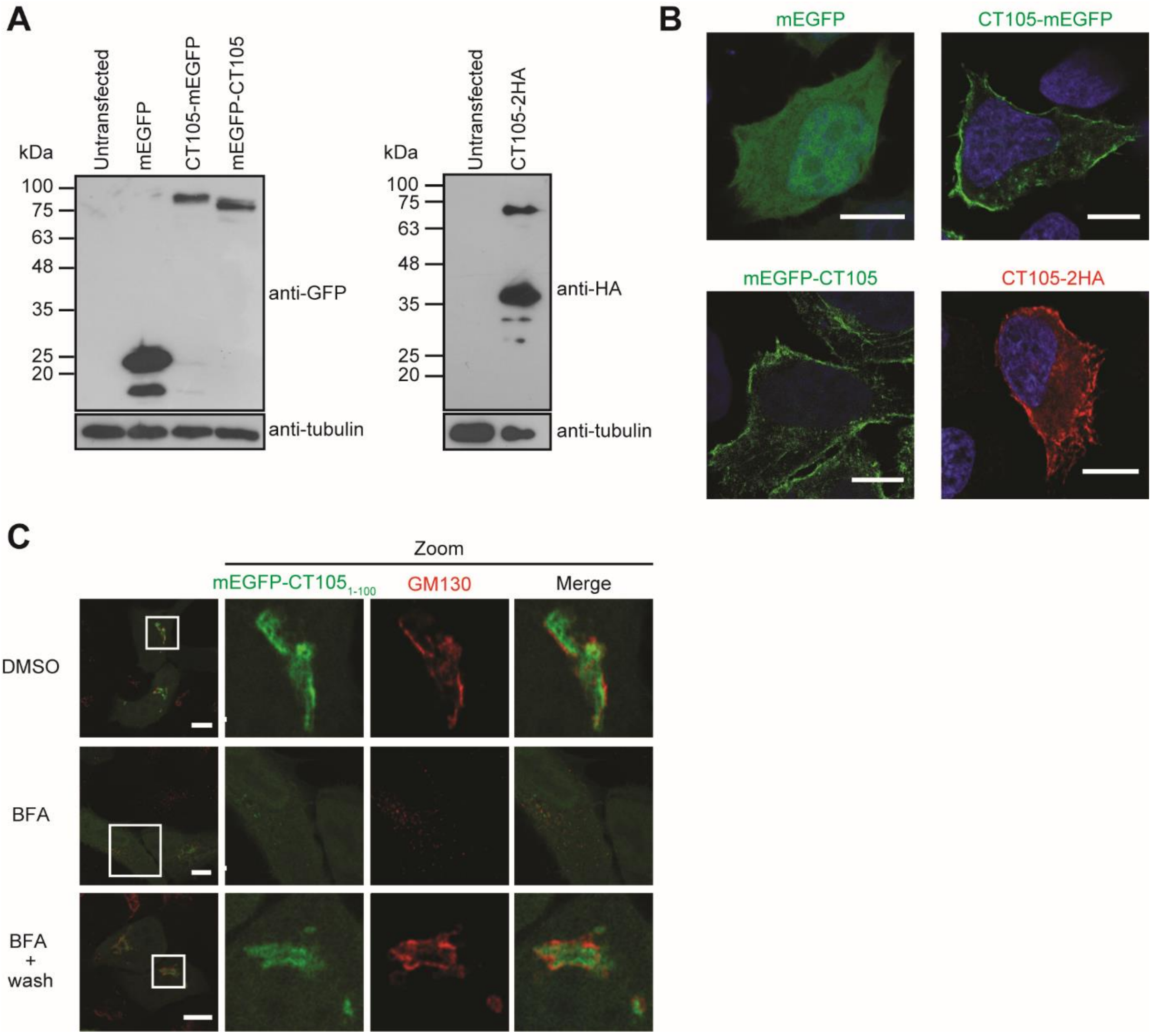

SUPPLEMENTARY FIGURE S9. Ectopic expression of CT105 in HeLa cells.
HeLa cells were either left untransfected or transfected for 24 h with plasmids encoding monomeric EGFP (mEGFP), CT105 fused to the C-terminus of mEGFP (mEGFP-CT105), CT105 fused to the N-terminus of mEGFP (CT105-mEGFP), CT105 with a double hemagglutinin tag at its C-terminus (CT105-2HA), or the first 100 amino acids of CT105 fused to the C-terminus of mEGFP (mEGFP-$CT105_{1-100}$)

A. Total extracts of the indicated untransformed or transfected cells were analyzed by immunoblotting with antibodies against GFP, HA and α-tubulin (HeLa loading control) using SuperSignal West Pico detection kit (Thermo Fisher Scientific).

B. Cells were fixed with 4% (w/v) paraformaldehyde (PFA), stained with 4',6-diamidino-2-phenylindole (DAPI; blue), and immunolabeled with an antibody against HA (red) and the appropriate fluorophore-conjugated secondary antibody.

C. Cell were treated with dimethyl sulfoxide (DMSO) or 1 µg/ml Brefeldin A (BFA) for 1 h. The cells were then either fixed with 4% (w/v) PFA or washed with medium lacking BFA and incubated for an additional 1h until fixation with 4% (w/v) PFA. Fixed cells were immunolabelled with antibodies against GM130 (red), and the appropriate fluorophore-conjugated secondary antibody

Cells were imaged by confocal fluorescence microscopy and images correspond to single *z* sections. Scale bars, 10 µm.

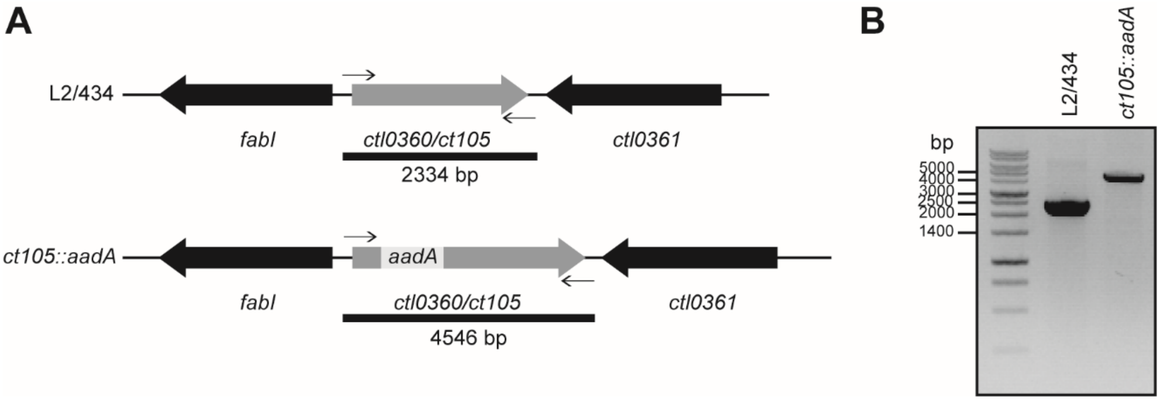

SUPPLEMENTARY FIGURE S10. Verification of intron insertion in the *C. trachomatis ct105*::*aadA* mutant strain.

A. Representation of the *ctl0360* (orthologue of c*t105* in strain D/UW3) locus in *C. trachomatis* L2/434 and of the *ctl0360* locus in the corresponding *ct105:aadA* mutant derivative. The arrows indicate the approximate hybridization position of the DNA primers used (Supplementary Table S4) in PCR reactions, yielding DNA products of the indicated length in base pairs (bp).

B. Agarose gel displaying the result from the PCR with the indicated primers (Supplementary Table S4) and DNA templates.

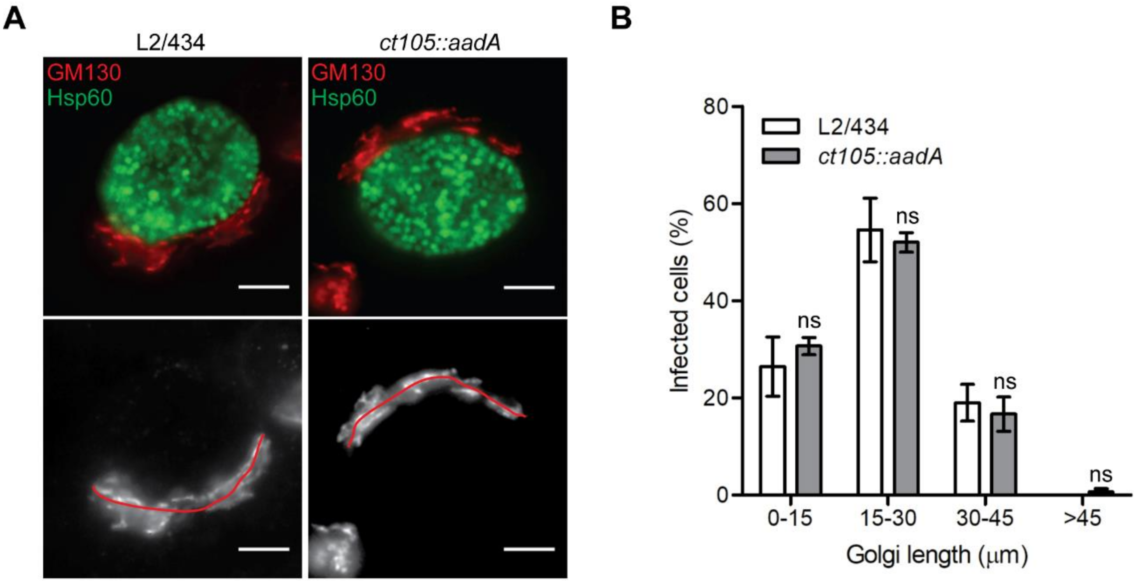

SUPPLEMENTARY FIGURE S11. The CT105 *Chlamydia trachomatis* effector protein does not affect host cell Golgi morphology during infection.

HeLa cells were infected by *C. trachomatis* L2/434 or *ct105::aadA* mutant for 24 h, fixed with methanol, and then immunolabeled with antibodies against GM130 (a *cis*-Golgi marker; red) and chlamydial Hsp60 (green), and appropriate fluorophore-conjugated secondary antibodies. **A.** Cells were imaged by fluorescence microscopy. The length of the Golgi complex around the inclusion was measured as described [13], which is illustrated by the red line. Scale bars, 5 µm.

B. Percentage of cells infected by *C. trachomatis* with Golgi around the inclusion of the indicated length. Data is average ± standard error of the mean from 3 independent experiments where at least 50 infected cells were analyzed for each strain. P-values were calculated by a two-tailed unpaired Student's *t*-test; ns, not significant ($P > 0.05$).

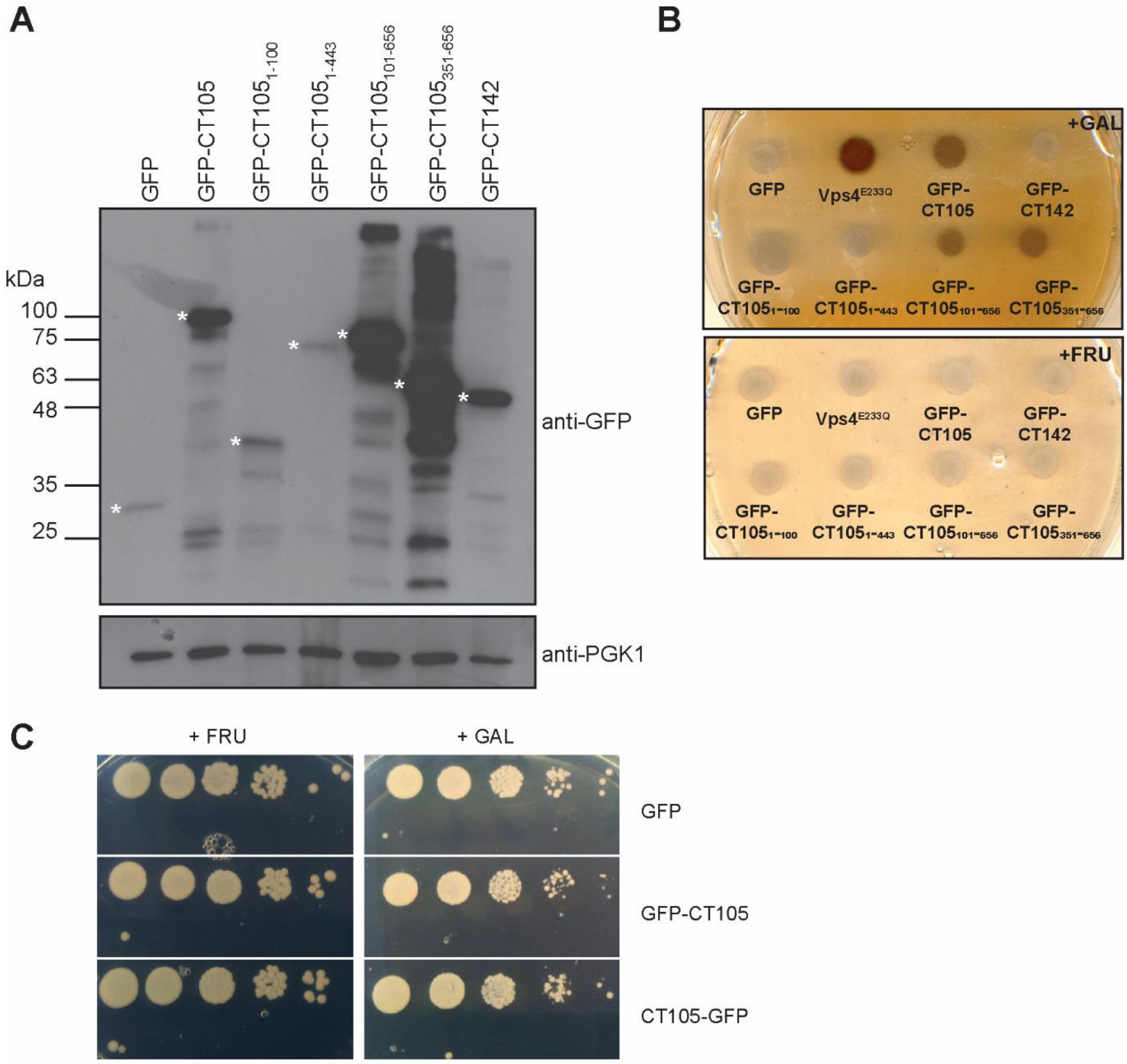

SUPPLEMENTARY FIGURE S12. The C-terminal region of CT105 induces a vacuolar protein sorting defect in *Saccharomyces cerevisiae* that is not related to yeast toxicity.

S. cerevisiae reporter strain NSY01 producing CPY-invertase was transformed with plasmids encoding GFP (pYES2-GFP; 30 kDa), GFP-CT105 (95 kDa), GFP-$CT105_{1\text{-}100}$ (37 kDa) GFP-$CT105_{1\text{-}443}$ (72 kDa), GFP-$CT105_{101\text{-}656}$ (85 kDa), $CT105_{351\text{-}656}$ (61 kDa), or GFP-CT142 (58 kDa), where their expression can be induced by galactose. A NSY01 derivative strain encoding a dominant-negative form of the yeast ATPase Vps4 ($Vps4^{E233Q}$) was also used. Yeast strains are listed in Supplementary Table S2.

A. Whole cell extracts of *S. cerevisiae* NSY01 producing the indicated GFP-fusion proteins were analyzed by immunoblotting with antibodies against GFP and PGK1 (yeast loading control), using SuperSignal West Pico detection kit (Thermo Fisher Scientific). Bands corresponding to the predicted molecular mass are indicated with a white asterisk.

B. *S. cerevisiae* NSY01 strains encoding the indicated proteins were grown in solid medium in the presence of galactose (inducing conditions, +GAL) or in the presence of fructose (non-inducing conditions; +FRU). The vacuolar protein sorting (VPS) phenotype was analyzed using a sucrose overlay to assess activity of secreted invertase. A vacuolar protein sorting defect (VPS^-) leads to the formation of a brown precipitate.

C. *S. cerevisiae* NSY01 harboring plasmids encoding GFP, GFP-CT105, or CT105-GFP were grown to late log phase in 2% (w/v) glucose. Then, 10-fold dilutions were spotted onto agar plates supplemented with 2% (w/v) fructose (non-inducing conditions; +FRU) or 2% (w/v) galactose (inducing conditions; +GAL) and incubated at 30°C for 3 days.

SUPPLEMENTARY REFERENCES (all listed in the main document)

1 Li, Z. *et al.* Characterization of fifty putative inclusion membrane proteins encoded in the Chlamydia trachomatis genome. *Infect Immun* **76**, 2746-2757 (2008).

2 Gehre, L. *et al.* Sequestration of host metabolism by an intracellular pathogen. *eLife* **5**, e12552, doi:10.7554/eLife.12552 (2016).

3 Shohdy, N., Efe, J. A., Emr, S. D. & Shuman, H. A. Pathogen effector protein screening in yeast identifies Legionella factors that interfere with membrane trafficking. *Proc Natl Acad Sci U S A* **102**, 4866-4871, doi:10.1073/pnas.0501315102 (2005).

4 Franco, I. S., Shohdy, N. & Shuman, H. A. The Legionella pneumophila Effector VipA Is an Actin Nucleator That Alters Host Cell Organelle Trafficking. *PLoS Pathog* **8**, e1002546, doi:10.1371/journal.ppat.1002546 (2012).

5 Bauler, L. D. & Hackstadt, T. Expression and targeting of secreted proteins from Chlamydia trachomatis. *J Bacteriol* **196**, 1325-1334, doi:10.1128/JB.01290-13 (2014).

6 Agaisse, H. & Derre, I. A C. trachomatis cloning vector and the generation of C. trachomatis strains expressing fluorescent proteins under the control of a C. trachomatis promoter. *PLoS ONE* **8**, e57090, doi:10.1371/journal.pone.0057090 (2013).

7 da Cunha, M. *et al.* Identification of type III secretion substrates of Chlamydia trachomatis using Yersinia enterocolitica as a heterologous system. *BMC Microbiol* **14**, 40, doi:10.1186/1471-2180-14-40 (2014).

8 Falcon-Perez, J. M., Nazarian, R., Sabatti, C. & Dell'Angelica, E. C. Distribution and dynamics of Lamp1-containing endocytic organelles in fibroblasts deficient in BLOC-3. *J Cell Sci* **118**, 5243-5255, doi:10.1242/jcs.02633 (2005).

9 Hammond, G. R. *et al.* PI4P and PI(4,5)P2 are essential but independent lipid determinants of membrane identity. *Science* **337**, 727-730, doi:10.1126/science.1222483 (2012).

10 Key, C. E. & Fisher, D. J. Use of Group II Intron Technology for Targeted Mutagenesis in Chlamydia trachomatis. *Methods Mol Biol* **1498**, 163-177, doi:10.1007/978-1-4939-6472-7_11 (2017).

11 Rodriguez-Escudero, I. *et al.* Reconstitution of the mammalian PI3K/PTEN/Akt pathway in yeast. *Biochem J* **390**, 613-623, doi:10.1042/BJ20050574 (2005).

12 de Felipe, K. S. *et al.* Legionella eukaryotic-like type IV substrates interfere with organelle trafficking. *PLoS Pathog* **4**, e1000117, doi:10.1371/journal.ppat.1000117 (2008).

13 Wesolowski, J. *et al.* Chlamydia Hijacks ARF GTPases To Coordinate Microtubule Posttranslational Modifications and Golgi Complex Positioning. *mBio* **8**, doi:10.1128/mBio.02280-16 (2017).
